# A long-term study on structural changes in calcium aluminate silicate hydrates

**DOI:** 10.1617/s11527-022-02080-x

**Published:** 2022-11-24

**Authors:** Sonya Barzgar, Yiru Yan, Mohamed Tarik, Jorgen Skibsted, Christian Ludwig, Barbara Lothenbach

**Affiliations:** 1 Empa, Concrete & Asphalt Laboratory, CH-8610 Dübendorf, Switzerland; 2 École Polytechnique Fédéral de Lausanne (EPFL), ENAC IIE GR-LUD, CH-1015 Lausanne, Switzerland; 3 Paul Scherrer Institute (PSI), ENE LBK CPM, 5232 Villigen PSI, Switzerland; 4 Aarhus University, Department of Chemistry and Interdisciplinary Nanoscience Center, 8000 Aarhus C, Denmark; 5 NTNU, Department of Structural Engineering, Trondheim, Norway; 6 Present Address: Sweco UK, Advisory and Planning Division, EC2M 7LS London, UK

**Keywords:** CO_2_ emission, Blended cement, C–A–S–H, Aluminum, Equilibration time, NMR

## Abstract

Production of blended cements in which Portland cement is combined with supplementary cementitious materials (SCM) is an effective strategy for reducing the CO_2_ emissions during cement manufacturing and achieving sustainable concrete production. However, the high Al_2_O_3_ and SiO_2_ contents of SCM change the chemical composition of the main hydration product, calcium aluminate silicate hydrate (C–A–S–H). Herein, spectroscopic and structural data for C–A–S–H gels are reported in a large range of equilibration times from 3 months up to 2 years and Al/Si molar ratios from 0.001 to 0.2. The ^27^Al MAS NMR spectroscopy and thermogravimetric analysis indicate that in addition to the C–A–S–H phase, secondary phases such as strätlingite, katoite, Al(OH)_3_ and calcium aluminate hydrate are present at Al/Si ≥ 0.03 limiting the uptake of Al in C–A–S–H. More secondary phases are present at higher Al concentrations; their content decreases with equilibration time while more Al is taken up in the C–A–S–H phase. At low Al contents, Al concentrations decrease strongly with time indicating a slow equilibration, in contrast to high Al contents where a clear change in Al concentrations over time was not observed indicating that the equilibrium has been reached faster. The ^27^Al NMR studies show that tetrahedrally coordinated Al is incorporated in C–A–S–H and its amount increases with the amount of Al present in the solution.

**Supplementary Information:**

The online version contains supplementary material available at 10.1617/s11527-022-02080-x.

## Introduction

Portland cement (PC) is produced by heating limestone and clay or other calcium carbonate and silicate mixtures at high temperatures (~ 1450 °C) in a rotary kiln. During this process limestone (CaCO_3_) breaks down to calcium oxide (CaO) and carbon dioxide (CO_2_) [1–3]. The CO_2_ emissions associated with cement production is around 800 kg per metric tonne of manufactured cement [1, 4]. Reducing these CO_2_ emissions is thus an urgent and important need [4, 5]. Using ‘carbon capture and storage (CCS)’ technologies in cement production for reducing the CO_2_ emissions is becoming attractive, although it is not cost-effective. Another approach focuses on the use of other binder types such as alkali activated binders or geopolymers, as replacement for Portland cement in the concrete composition, although their long-term durability remain unclear [5]. Furthermore, the use of different waste materials as fuel is also an opportunity to reduce the CO_2_ intensity with expanding the use of biomass and alternative fuels, however, the maximum reduction in CO_2_ emissions which can be achieved by sustainable fuels is only approx. 40% of the total CO_2_ emission from Portland cement production, since about 60% originates from the decarbonation of limestone [6]. A well-established strategy to reduce the emission of CO_2_ is to lower the clinker content by partial replacement of Portland cement with supplementary cementitious materials (SCM) such as limestone, blast furnace slags, by-products from steel production, fly ash, from coal combustion, or calcined clays [3, 7–10]. The addition of SCM results in cements which are now widely considered superior to conventional cement, and that aids in expanded production capacity, reducing the amount of energy used and CO_2_ emissions during the calcination process [2, 3].

Calcium silicate hydrate (C–S–H) phase is the main hydration product during Portland cement hydration, which is responsible for a large part of the cohesive properties of cement [5, 7, 11]. Replacement of PC with SCM largely affects the chemistry of the binder gel as significant amounts of aluminum (Al) and silicon (Si) can be incorporated into the primary reaction product, C–S–H, which influences its structure and composition by e.g. reducing its Ca/Si ratio [7]. Aluminum can be taken up in the C–S–H structure by substituting the bridging silicon in the silicate dreierketten units, which results in Al substituted C–S–H also known as “C–A–S–H” [12–18]. The C–S–H and C–A–S–H structure can be related to a defect tobermorite structure, which contains polyhedral layers of calcium oxide linked on both sides to “dreierketten”—tetrahedral (alumino) silicate chains with a repeating pattern every three tetrahedra [12, 18–21]. Two of the three tetrahedra are linked to the calcium oxide layer. The third tetrahedron, the bridging tetrahedron, connects the dimer of pairing tetrahedra to the next dimer [15, 18, 22, 23]. These silica chains have a variable length, which are dependent on the Ca/Si ratio [24, 25]. The counter-ions (e.g., Ca^2+^, Na^+^ and OH^−^) and water are present in the interlayer [22, 23, 26, 27]. At a Ca/Si ratio of 0.67, calcium ions are not present in the interlayer and the bridging tetrahedra connect the dimers in the silicate chains [18, 28]. At high Ca/Si ratios, the high Ca content in the interlayer results in shorter silica tetrahedral chains [29]. Aluminum is incorporated in the bridging sites of silica tetrahedral chains [23, 30] in C–S–H and it may occur in tetrahedral, pentahedral or octahedral coordination for C–A–S–H samples with high Ca/Si ratios [31].

The effect of aluminum uptake on the structure of C–S–H is not yet completely understood. A number of studies investigated the effect of varying Al/Si ratio on the structure of C–A–S–H gel [13, 32, 33]. Experimental investigations showed an increase in the amount of incorporated Al in C–S–H with increasing the aluminum concentrations in solution [14, 16, 29, 34–38]. The precipitation of secondary phases such as microcrystalline aluminum hydroxide (Al(OH)_3_), katoite (3CaO·Al_2_O_3_·6H_2_O) and strätlingite (2CaO·Al_2_O_3_·SiO_2_·8H_2_O) limits the concentration of aluminum in solution as well as the Al uptake in C–S–H [14, 16, 30, 34, 37]. Previous experimental studies on Al sorption in C–S–H have not covered the whole range of Al contents, they concentrated either on Al uptake in C–S–H at relatively high Al content (Al/Si ≥ 0.05) [13, 15, 29, 33] or at lower Al contents (Al/Si from 0.001 to 0.1) [35].

In this study, sorption isotherms over the entire range of Al/Si ratios from 0.001 to 0.2 are presented to investigate how pH and equilibration time affect the uptake of Al in the C–S–H as well as in the structure of C–A–S–H. The gained knowledge on the aqueous and solid phase composition of C–A–S–H is needed for further development of thermodynamic models describing the Al uptake in C–S–H, used for calculating the C–A–S–H composition in hydrating cements [39, 40].

## Material and methods

The effect of Al concentrations on Al uptake in C-S–H was studied by performing long-term sorption isotherm experiments. The synthesis of samples was followed by the same procedure, as detailed in [35, 38]. A total of 4 g calcium oxide (CaO), silica fume (SiO_2_, Aerosil 200, Evonik) and calcium aluminate (CA: CaO·Al_2_O_3_) with different proportion as detailed in Appendix F was added into 180 mL of Milli-Q water or sodium hydroxide (NaOH) solutions (liquid/solid = 45 mL/g) in order to obtain C–A–S–H with different compositions. Calcium carbonate (CaCO_3_, Merck, pro analysis) was heated at 1000 °C for 12 h to obtain CaO. A mixture of CaCO_3_ and aluminum oxide (Al_2_O_3_) (Sigma Aldrich) was heated at 800 °C for 1 h, at 1000 °C for 4 h and at 1400 °C for 8 h followed by cooling down with a rate of 600 °C/h [16] to synthesize CA. The investigations were performed on C–A–S–H samples with a target Ca/Si ratio of 0.8. In addition, different NaOH concentrations of 0, 0.1, 0.5 and 1 M were used to be able to cover the pH range of hydrated cements [29].

After synthesizing the samples in a nitrogen-filled glovebox, they were stored in 200 mL PE-HD containers. The containers were placed on a horizontal shaker moving at 100 rpm and equilibrated for different times at 20 °C. The solid and liquid phases were separated by vacuum filtration using nylon filters (pore size: 0.45 μm) and analyzed.

### Solution phase analysis

The elemental concentrations of Na, Ca, Si and Al in the filtrates were measured with Inductively Coupled Plasma Mass Spectrometry (ICP-MS; Agilent 7700x), Inductively Coupled Plasma Optical Emission Spectrometry (ICP-OES; Spectro Arcos) and/or Ion Chromatography (IC). IC was used to measure concentrations of sample with Al/Si > 0.05. ICP-MS and ICP-OES were used to measure samples at lower Al concentrations as the Al concentrations were very low and these two instruments provide results with much lower limit of detection compared to IC. The experimental results were validated using different techniques. Samples analyzed by ICP-MS and ICP-OES were first acidified to contain 1% HNO_3_ (using Suprapur HNO_3_, Merck). In samples containing NaOH, the Na concentrations were kept below 230 mg/L and 1100 mg/L for the ICP-MS and ICP-OES analysis, respectively by further diluting the samples. The blank solution (1% HNO_3_) and multi-standard solutions were prepared in such a way to contain all the elements, Al, Ca and Si, in the range from 0 to 20 mg/L and from 0 to 200 µg/L for ICP-OES and ICP-MS, respectively. In alkali-free samples, the standard solutions were prepared containing Ca, Si and Al. However, in samples containing NaOH, 1500 mg/L and 230 mg/L of Na were added to the standard solutions for the ICP-OES and ICP-MS analysis, respectively. The goal of this procedure was to minimize the matrix effect and ascertain that all samples including the standard solutions have the same Na concentration.

The samples with a target Al/Si of 0.05–0.2 synthesized in 0.1, 0.5 and 1 mol/L NaOH were analysed by IC as soon as possible after filtration, and diluted by factor 10, 100 and 1000 with MilliQ water to avoid any carbonation or/and precipitation. A Dionex DP series ICS-3000 ionic chromatography system was used to quantify the concentration of Ca, Na, Al and Si.

The OH^−^ concentration of the not-diluted samples was measured by conducting the pH measurements using a Knick pH meter (pH-Meter 766) equipped with a Knick SE100 electrode at room temperature (T). The calibration of pH electrode was made against 0.1, 0.2, 0.5 and 1 M NaOH solutions for reducing the alkali error as detailed in [41].

### Solid phase analysis

After the filtration, C–A–S–H samples were first washed with a 50–50% (volumetric) water–ethanol solution in order to avoid the precipitation of alkali salts during drying. Then, the samples were washed with 94% ethanol solution inside the glovebox to get rid of any free water. Afterwards, the samples were dried in a freeze dryer for almost one week followed by storage in nitrogen filled desiccators containing saturated CaCl_2_·2H_2_O solution, generating a relative humidity of 30% [14, 42].

The composition and structure of the solid phase were further studied by using different techniques such as Thermogravimetric Analysis (TGA), Fourier-Transform Infrared Spectroscopy (FTIR) and ^27^Al Magic Angle Spinning Nuclear Magnetic Resonance (MAS-NMR) spectroscopy. TGA data were obtained from the TGA/SDTA851e Mettler Toledo device using a heating rate of 20 °C/min. The weight loss of approximately 30 mg of sample was recorded under N_2_ atmosphere in a temperature range of 30 °C up to 980 °C. The weight loss between 150–220, 220–300, 300–350, 350–450 and 600–800 °C were associated with strätlingite, Al(OH)_3_, katoite, portlandite and CaCO_3_, respectively, and determined using the tangential method [43]. The amount of these solids was calculated based on the theoretical weight loss of these solids.

FTIR spectra were measured on powder from 600 to 4300 cm^−1^ on a Bruker Tensor 27 spectrometer with a resolution of 4 cm^−1^ by transmittance. In order to make the comparison easier, the spectra were first background corrected and then scaled to the maximum signal of Si–O bond vibrations at 1100 cm^−1^.

The ^27^Al MAS NMR spectra were recorded on a Varian Direct-Drive VNMR-600 spectrometer (14.09 T) using a home-built CP/MAS probe for 4 mm o.d. zirconia rotors and ν_R_ = 13.0 kHz, a recycle delay of 2 s, and 4096 scans. A short excitation pulse of τ_p_ = 0.5 µs for an rf field strength of γB1/2π = 60 kHz was employed. This ^29^Si MAS NMR spectra were obtained on a Bruker Avance HD spectrometer (9.39 T), using a 4 mm ^1^H-X probe, a spinning speed of ν_R_ = 10.0 kHz, a 3 µs excitation pulse (45° for a γB_1_/2π = 42 kHz), a recycle delay of 30 s, and + 2700 scans. The ^27^Al and ^29^Si MAS NMR spectra were referenced to 1.0 M AlCl_3_·6H_2_O and neat TMS, respectively.

### Thermodynamic modeling

Thermodynamic modeling was performed to derive the saturation indexes in solution with respect to different solids, which could potentially form, using the version 3.7 of the Gibbs Free Energy Minimization (GEM-Selektor) software [44]. Thermodynamic data for portlandite, amorphous SiO_2_ and aqueous species were selected from the PSI-Nagra thermodynamic database [45], the solubility of strätlingite, microcrystalline Al(OH)_3_, C–S–H and katoite from the Cemdata18 database [46] and the solubility of the zeolites Ca-gismondine (CaAl_2_Si_2_O_8_·4.5H_2_O), OH-sodalite (Ca_8_Al_6_Si_6_O_24_(OH)_2_·2H_2_O) and chabazite (CaAl_2_Si_4_O_12_·6H_2_O) from [47, 48]. The CSHQ thermodynamic solid solution model was used to model the Ca and Si concentrations in the C-S–H system [49]. The measured total elemental concentrations of Ca, Si, and Al and of OH^−^ from pH measurements were used to calculate saturation indices to assess the potential formation of secondary phases present in the solid at different conditions of the system (pH value, equilibration time, …) as discussed in detail in [16]. The activity coefficients of the aqueous species ${\gamma }_{i}$ were calculated using the extended Debye-Hückel equation (Eq. 1) with common ion-size parameter ${a}_{i}$ = 3.31 Å for NaOH solutions [50] and common third parameter *b*_*y*_ according to:

1 $$\log \gamma_{i} = \frac{{ - A_{y} z_{i}^{2} \sqrt I }}{{1 + B_{y} a_{i} \sqrt I }} + b_{y } I$$

where *I* denotes the effective molal ionic strength, ${z}_{i }$ the charge of species $i,$ *b*_*y*_ is a semi-empirical parameter (∼0.098 for NaOH electrolyte at 25 °C), and ${A}_{y}$ and ${B}_{y}$ are $P,T$-dependent coefficients. The Debye-Hückel activity correction is appropriate up to ∼1 M ionic strength [51].

Saturation indices (SI) were calculated based on Eq. 2 using GEMS. The ion activity product (*IAP*) was calculated using the concentrations of Al, Ca, Si and Na in solution and the measured pH values. The *K*_*so*_ represents the theoretical solubility product of the respective solid. A negative saturation index (< 0) indicates undersaturation, while a positive value indicates the oversaturation and possibly precipitation of solid phase. The SI calculation was used to independently assess which solids can potentially form.

2 $$SI = \log \left( {\frac{IAP}{{K_{so} }}} \right)$$

### Al uptake in C–S–H

The incorporation of Al in the C–A–S–H phases was not only obtained by ^27^Al MAS NMR but also estimated by mass-balance calculations. The quantification of the secondary phases was performed by TGA and to calculate the effective C–A–S–H composition, the amount of Si, Ca, Al and Na in secondary phases and the fraction of Al, Ca, Si and Na in solution were subtracted from the initial quantities as explained in details in [16, 35, 38]. For example, for the calculation of molar Al/Si ratio in C–A–S–H, the mass of Al in secondary phases containing Al (strätlingite, Al(OH)_3_, katoite) and the mass of Al in solution were subtracted from the initial mass of Al in CaO·Al_2_O_3_ used in synthesis. The same method was followed to calculate the mass of Si in C–A–S–H. Then, the molar Al/Si ratio was calculated using the molar quantity of Al and Si in C–A–S–H. The detail of the measurements and quantifications are presented in Appendix A. The measurement errors were taken into account in the calculation of elemental compositions in C–A–S–H. The errors of concentrations in the aqueous solution are less than 2%. An additional error of 10% was considered in the quantification of different secondary phases with TGA. The calculated measurement errors in Al/Si and Ca/Si ratios for the C–A–S–H samples are compiled in Appendix D.

The uptake of Al into C–S–H phases is expressed in terms of a *K*_*d*_ value (distribution coefficient), which equals to the ratio of the quantity of aluminum adsorbed per unit mass of solid to the quantity of aluminum remaining in solution at equilibrium. The *K*_*d*_ values were calculated according to Eq. 3:

3 $$K_{d} = \frac{{C_{s,eq} }}{{C_{l,eq} }}\left( {m^{3} /kg} \right)$$

where *C*_*s,eq*_ is the equilibrium concentration of Al being sorbed on the C–A–S–H phases (mol/kg) and *C*_*l,eq*_ is the equilibrium concentration in solution (mol/m^3^) [52]. The errors of *K*_*d*_ values are generally less than 1% as compiled in detail in Appendix D.

## Results and discussion

### C–A–S–H without NaOH

#### The effect of Al concentration on the formation of secondary phases

**Fig. 1:**
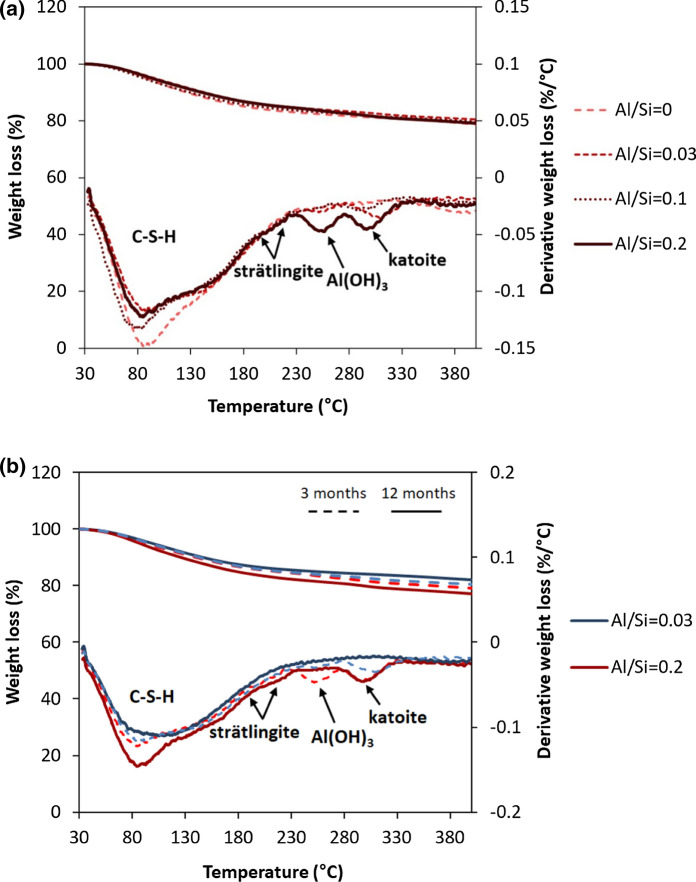
The effect of** a** Al content after 3 months equilibration and** b** equilibration time on secondary phases' content for target Ca/Si = 0.8 in the absence of NaOH

 Figure 1 shows the effect of the Al/Si ratios and the equilibration time on solid phases formed, determined from the TGA analysis of C–A–S–H samples with target Ca/Si = 0.8 in the absence of NaOH after 3 and 12 months equilibration. C–A–S–H is in all cases the main hydrate formed. At low Al/Si ratios, only C–A–S–H is present, however, at higher Al/Si ratios (≥ 0.03) secondary phases such as strätlingite, katoite, portlandite and Al(OH)_3_ also precipitate. Increasing the target Al/Si ratio from 0.03 to 0.2 increases the content of aluminum hydroxide and katoite from 0.29 wt% and 1.7 wt% to 1.4 wt% and 2.1 wt%, respectively (Fig. 1a). Similarly, the presence of katoite and strätlingite at target Al/Si ≥ 0.1 and Al(OH)_3_ at target Al/Si = 0.33 has been observed [16, 30, 35]. Details on the amounts of secondary phases are given in the Supporting Information, Appendix A.

 Fig. 1b illustrates how longer equilibration times of 12 months decrease the content of Al(OH)_3_ and katoite. Al(OH)_3_ is only present after 3 months equilibration at target Al/Si ratios of 0.03 to 0.2. The content of katoite decreases from 1.7 wt% to an undetected level at target Al/Si ratio of 0.03 between 3 and 12 months. A similar complete destabilization of Al(OH)_3_ with time is observed at target Al/Si ratios of 0.1 and 0.2; only at target Al/Si = 0.2 some katoite is still present after 12 months. The disappearance of Al(OH)_3_ and katoite with time is consistent with the undersaturation observed for these solids in the solution (Appendix C) both after 3 and 12 months, which implies an initial precipitation of these solids followed by a slow dissolution with time. Similarly, L'Hôpital et al. [16] observed the persistence of katoite in the presence of C–A–S–H phases at Ca/Si = 1.0 after 6 months equilibration although the solution was strongly undersaturated. Similar behavior have also been shown for brucite in the presence of M–S–H (magnesium silicate hydrate); the kinetic hindrance of brucite dissolution was related to the presence of Si in solution [53]. It can be speculated that Si could slow down also Al(OH)_3_ and katoite dissolution at high pH values similarly to the slowdown of quartz dissolution in the presence of Al [54], although experimental evidence is presently missing.

The molar fraction of Al in C–A–S–H phases and in different secondary phases such as Al(OH)_3_, strätlingite and katoite for Ca/Si = 0.8 obtained from mass-balance is summarized in Appendix G. The Al(OH)_3_ and katoite are mainly present at target Al/Si ≥ 0.03 and strätlingite at target Al/Si = 0.2. At all NaOH concentrations, a higher Al/Si ratio leads to an increase in Al fraction bound in strätlingite, Al(OH)_3_ and katoite. In the absence of NaOH and after 3 months equilibration, an increase in Al/Si ratio from 0.001 to 0.2 leads to more Al(OH)_3_ and katoite from 0 to 10.7% and 6.4%, respectively. In the presence of 0.5 M NaOH, increasing the Al/Si ratio from 0.001 to 0.2 increases the Al fraction in katoite from 0 to 5.4% and 1.5% after 3 months and 15 months equilibration, respectively.

**Fig. 2:**
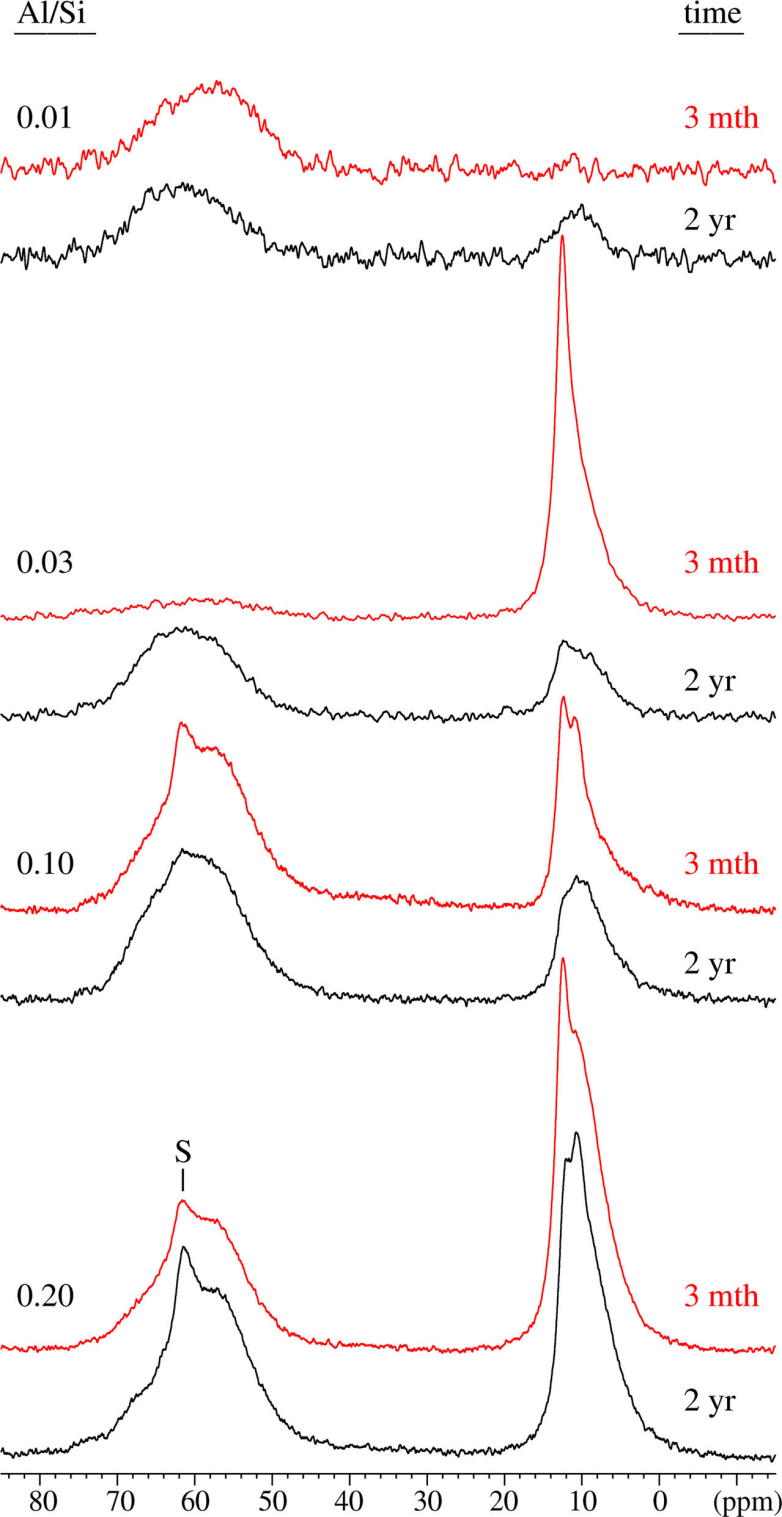
The ^27^Al MAS NMR spectra of the alkali-free C–A–S–H samples with target Al/Si ratios of 0.01 – 0.2 after equilibration times of 3 months and 2 years. The narrow Al(IV) resonance from strätlingite at 61 ppm is indicated by ‘S’

**Fig. 3:**
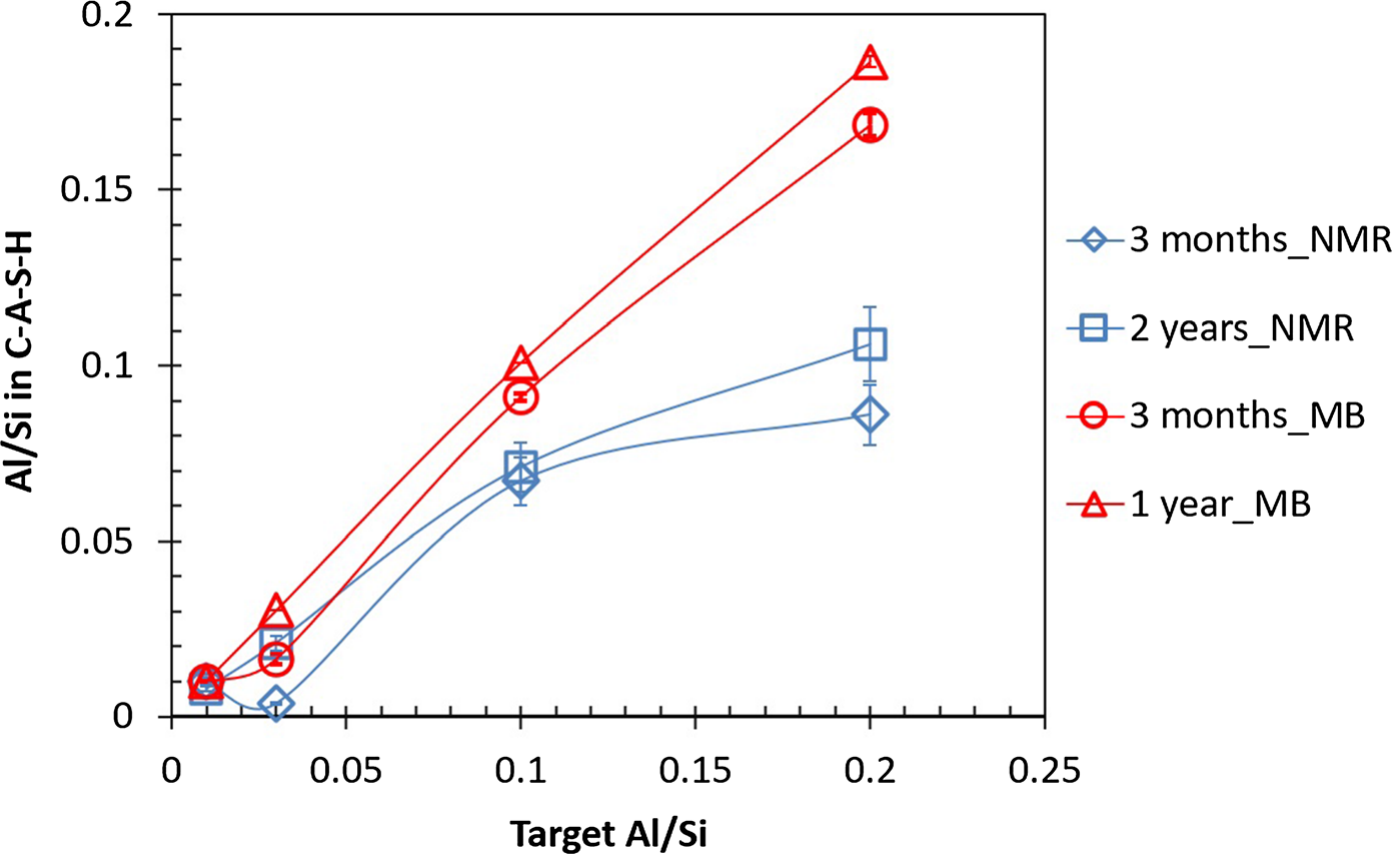
The molar Al/Si in C–A–S–H vs. target Al/Si ratios calculated using mass-balance based on TGA (MB) and ^27^Al MAS NMR. (The errors for mass-balance calculations are smaller than the symbols' size)

The nature of Al in selected alkali-free samples, where significant amounts of secondary phases were present, has been further investigated using solid-state ^27^Al MAS NMR spectroscopy as summarized in Table 1 and with the experimental spectra shown in Fig. 2. The results indicate also that the amount of Al in C–A–S–H phase increases with increasing Al/Si ratio, supporting the mass-balance results based on TGA. Again, at low target Al/Si of 0.01, all Al is found to be present in C–A–S–H, while at higher target Al/Si ratios (≥ 0.03) secondary phases such as katoite, strätlingite and most probably calcium aluminate hydrate (C–A–H) phases are also present. The six-fold coordinated aluminum (Al(VI)) resonances correspond to secondary phases such as katoite and C–A–H phases (monocarbonate, C_2_AH_8_, or CAH_10_). The absence of resonances at approx. 35 ppm and 5.0 ppm show that five-fold coordinated aluminum (Al(V)) or six-fold coordinated aluminum (Al(VI)) sites are not present in the C–S–H structure at Ca/Si = 0.8 in agreement with recent observations for C–A–S–H with varying Ca/Si ratios [31, 55]. It should be noted that although ^27^Al NMR and mass-balance calculations based on TGA show the same trends, the amount of secondary phases is strongly underestimated based on the TGA results as shown in Fig. 3. This is due to the difficulties in adequately deconvoluting the small, relatively broad shoulders caused by the presence of strätlingite or katoite in the presence of mainly C–S–H in TGA. The ^27^Al NMR data confirms that Al incorporated in C–S–H with target Ca/Si = 0.8 is tetrahedrally coordinated, following the assignment of the resonances in the range 50–75 ppm, which reflect different environments for Al(IV) in the silicate chains of the C–A–S–H structure, as recently investigated in detail [55]. From the intensities of the Al(IV) resonances, it is also apparent that the amount of Al(IV) in the C-S-H increases with increasing Al concentration.

**Table 1:** The Al speciation in the alkali-free C–A–S–H samples determined from solid-state ^27^Al MAS NMR for target Ca/Si = 0.8 after 3 months and 2 years equilibration at different Al/Si ratios

Target Al/Si	Time (months)	Al/Si in C–A–S–H	Al (IV)	% Al in C–A–S–H	Al (VI)	Secondary phases^a)^
0.01	3	0.01	1.00	100	–	–
0.03	3	0.0036	0.12	11.8	0.88	katoite, C–A–H
0.10	3	0.067	0.67	66.0	0.33	strätlingite, katoite, C–A–H, Al(OH)_3_
0.20	3	0.086	0.43	42.3	0.57	strätlingite, katoite, C–A–H, Al(OH)_3_
0.01	24	0.0079	0.79	79.0	0.21	katoite, C–A–H
0.03	24	0.021	0.70	70.0	0.30	katoite, C–A–H, Al(OH)_3_ peak/shoulder
0.10	24	0.071	0.71	71.0	0.29	katoite, C–A–H, Al(OH)_3_
0.20	24	0.106	0.53	52.9	0.47	strätlingite, katoite, C–A–H, Al(OH)_3_

Strätlingite is identified by its Al(IV) resonance at 61.5 ppm and katoite by the Al(VI) peak at 12.4 ppm. C–A–H may include CaAl_2_(OH)_8_·6H_2_O, Ca_2_Al_2_(OH)_10_·3H_2_O and the AFm phases Ca_4_Al_2_(OH)_14_·6H_2_O and Ca_4_Al_2_(OH)_12_CO_3_·5H_2_O (monocarbonate) which all have Al(VI) resonances in the range 10–11 ppm. The broad shoulder in the range 0–5 ppm is ascribed to alumina gel (Al(OH)_3_). Phases in parenthesis are present in very small amounts

**Fig. 4:**
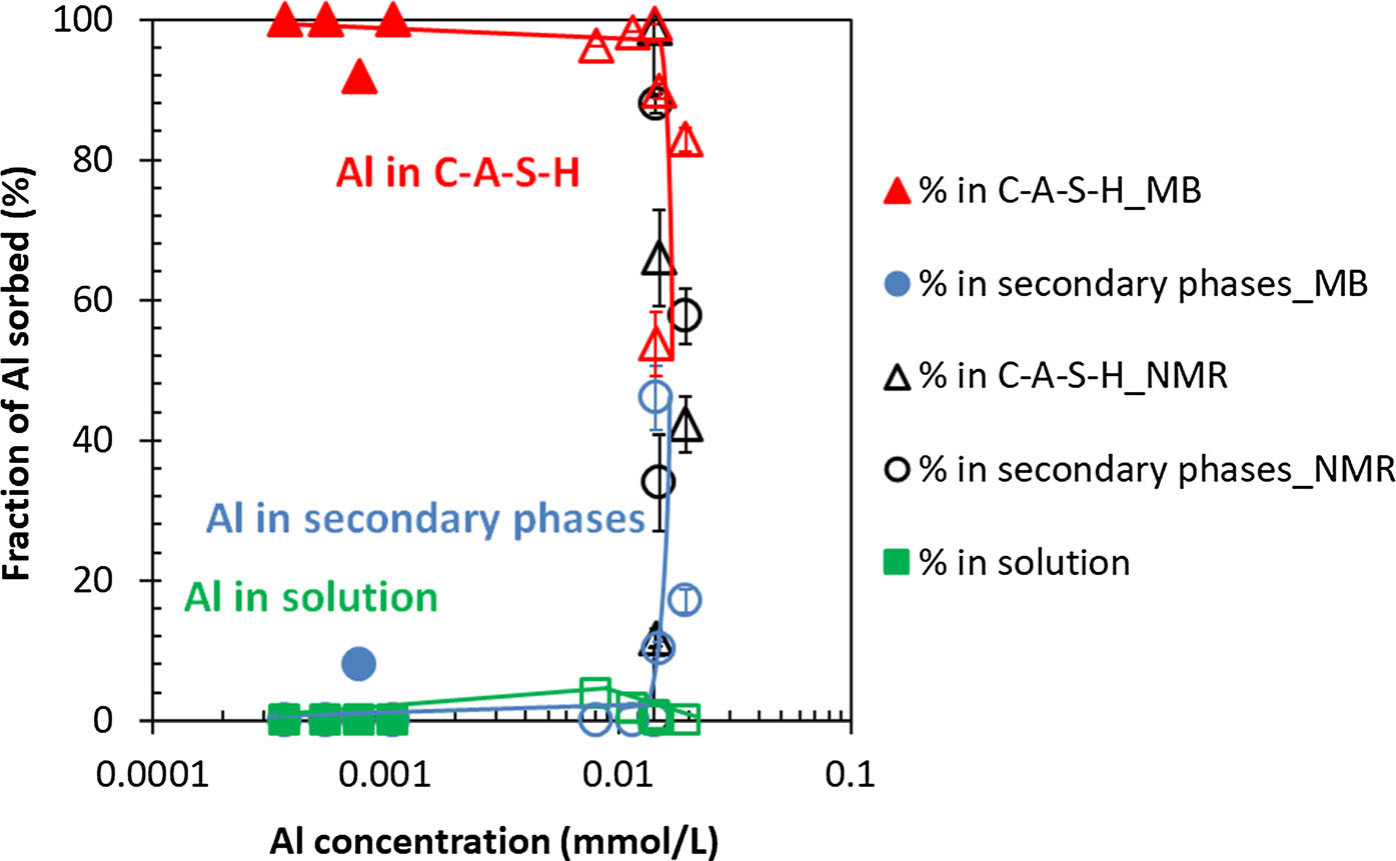
The Al fraction in solution, C–A–S–H and secondary phases as a function of the measured Al concentration for target Ca/Si = 0.8 in the absence of NaOH from mass-balance calculations based on TGA (MB) and ^27^Al MAS NMR results for 3 months (empty symbols) and from mass-balance calculations based on TGA after 12 months (filled symbols) equilibration. (The lines serve as eye-guides only and the errors for mass-balance calculations are smaller than the symbols' size)

At low Al contents, only the presence of C–A–S–H phase is observed, however, at higher Al contents secondary phases are formed in addition to the C–A–S–H phase. Figure 4 illustrates that increasing the Al concentrations leads to an increase in the fraction of Al in the secondary phases and to a decrease in Al fraction in C–A–S–H from nearly 100% at low Al content to about 50% at high Al/Si ratio. Even though a higher fraction of Al is present in secondary phases, the amount of Al in C-S–H increases as illustrated in Fig. 3. In fact, the ^27^Al MAS NMR studies indicate that an increase in target Al/Si ratio from 0.01 to 0.2 leads to a decrease in the fraction of Al in the C–A–S–H phase from 100% to 42.3%. Furthermore, mass-balance calculations point out that an increase in equilibration time from 3 to 12 months leads to a clear increase in the Al fraction in C–A–S–H and a decrease in Al content in the secondary phases. This is observed even at target Al/Si = 0.2 where 92% of the Al is present in C–A–S–H after 12 months; whereas after 3 months this value is 83%.

The effect of Al concentration on C–A–S–H structure.

FTIR and ^29^Si MAS NMR analysis have been performed in order to investigate the changes in the structure of C-S-H with different Al/Si ratios. The assignment of the adsorption bands is summarized in Table 2 and explained in detail in [38].

**Table 2:** Assignment of FTIR spectra for C–A–S–H samples

FTIR Absorption band (cm^−1^)	Assignment of vibration	References
500–750	Al–O stretching vibrations of octahedrally coordinated Al	[56]
661 and 906	Al–O stretching vibrations in Al(OH)_3_	[57]
665	Si–O–Si bending vibrations	[21, 58–62]
709, 710, 855, 860, 911, 913, 965, 970, 1016 and 1020	Si–O–Al in strätlingite	[63–65]
750–900	Vibrations of Al–O bond in AlO_4_ units	[56]
820	Si–O stretching of Q^1^ tetrahedra	[21, 58–62]
850–1300	Asymmetric and symmetric stretching vibration of Si–O-Si and Si–O–Al bonds in [SiO_4_]^4−^ and [AlO_4_]^5−^	[66–68]
880	Stretching vibration of Al–O–Si	[69]
900–1200	Stretching or bending vibration of Si–O bands (Q^1^ and Q^2^)	[70]
914–918	OH bending vibrations in Al–OH–Al bonds (octahedral aluminum)	[61]
1034–1075	Symmetric bending of Al–O–H	[57, 71, 72]

**Fig. 5:**
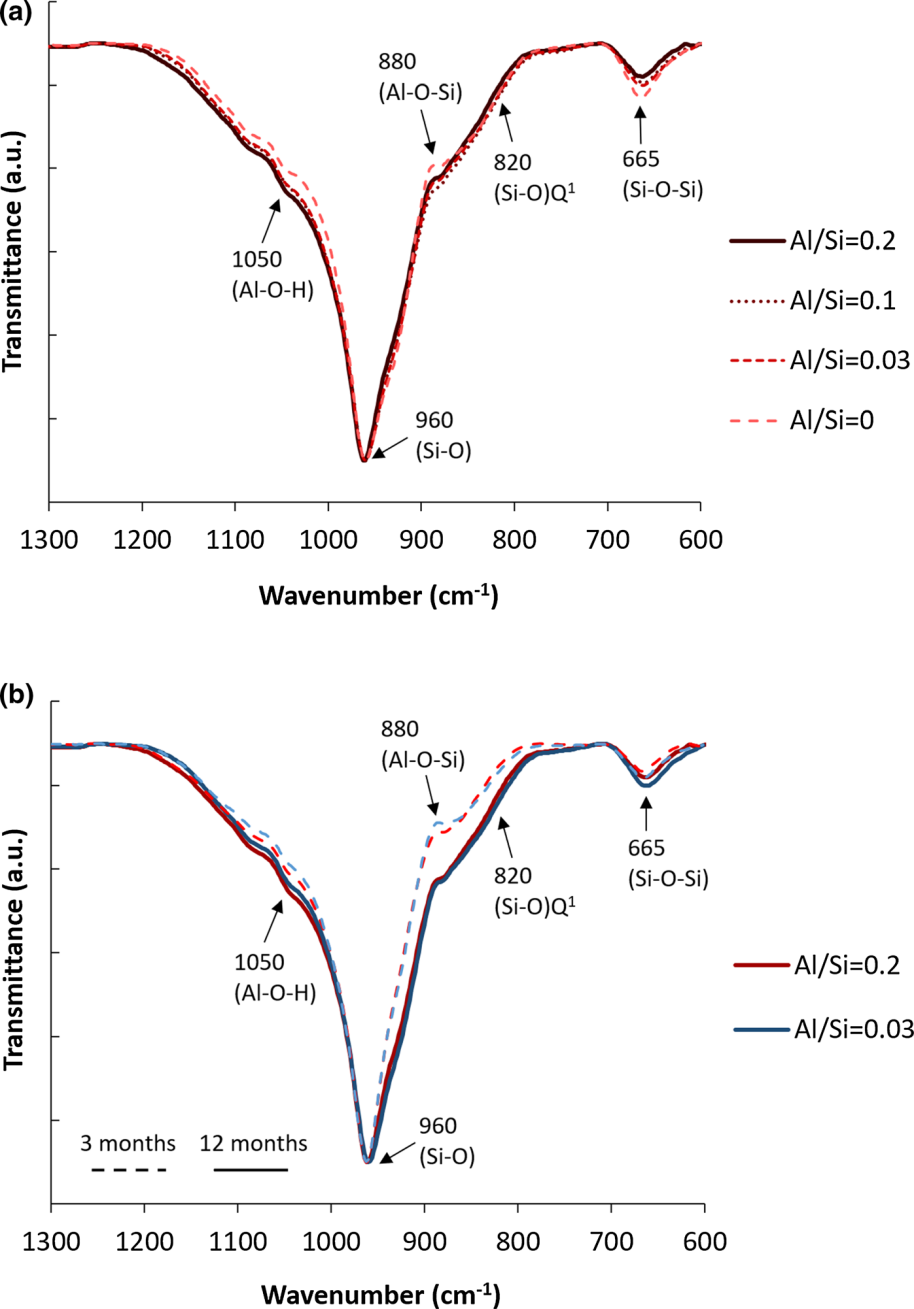
The FTIR spectra for C–A–S–H samples at target Ca/Si = 0.8 and in the absence of NaOH for** a** 12 months equilibration with different Al/Si ratios and** b** different equilibration times with target Al/Si ratios of 0.03 and 0.2

Figure 5 represents the FTIR spectra for the C-A-S-H samples synthesized (a) in the absence of NaOH for 12 months equilibration with target Al/Si ratios from 0 to 0.2 and (b) different equilibration times with target Al/Si ratios of 0.03 and 0.2. As shown in Fig. 5a, the increase in the intensity of the shoulder at 880 cm^−1^ indicates a higher Al uptake in C–A–S–H at higher Al contents. The band intensity at 665 cm^−1^ for Si–O–Si bending vibrations drops with increasing Al content, which could be related to the replacement of silica in the bridging position by Al. The intensity of the Si-O stretching vibration of Q^1^ tetrahedra at 820 cm^−1^ decreases at higher Al contents. Based on the ^27^Al and ^29^Si MAS NMR studies, Richardson and co-workers [18, 73] reported the disappearance of Q^1^ sites in low Ca/Si C–S–H in the presence of aluminum, which is also consistent with the substitution of Al(IV) only into the bridging tetrahedral sites of the dreierketten chains (Table). The shoulders at 1100 cm^−1^ and 1050 cm^−1^ could not be clearly assigned, although the intensity of those shoulder has been observed to increase with aluminum content in C–S–H samples equilibrated for 2 years [38].

**Fig. 6:**
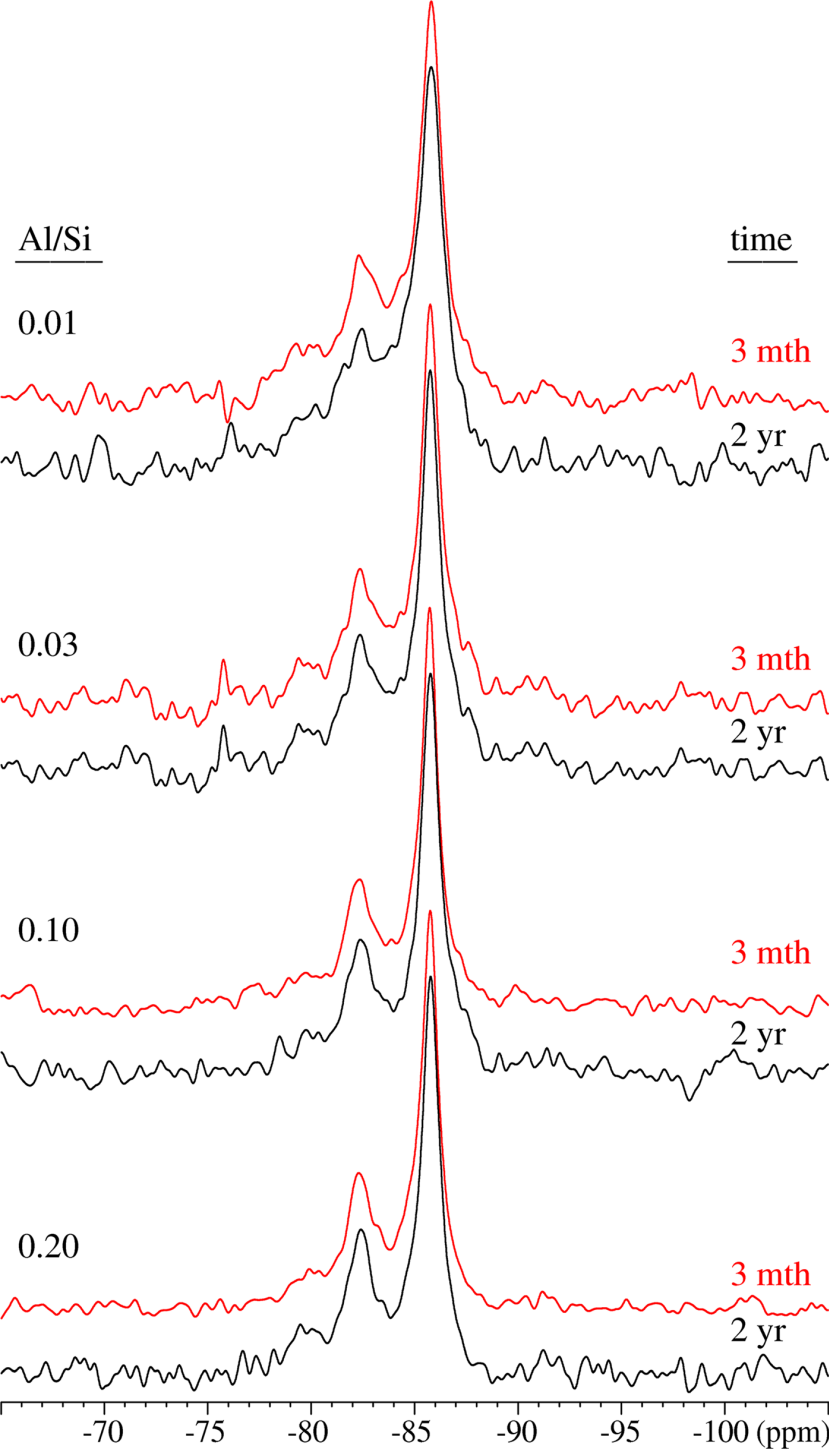
^29^Si MAS NMR spectra (9.39 T, ν_R_ = 10.0 kHz) of the alkali-free C–A–S–H samples with target Al/Si ratios of 0.01–0.2 after equilibration times of 3 months and 2 years

The FTIR spectra in Fig. 5b presents an increase in the intensity of the Al–O–Si shoulders at 880 cm^−1^ with longer equilibration time illustrating the presence of more Al in C–A–S–H. Also the intensity of the Q^1^ Si–O stretching vibration at 820 cm^−1^ and the shoulder at 920 cm^−1^ increases significantly with equilibration time for both target Al/Si ratios of 0.03 and 0.2 indicating a structural arrangement. The ^29^Si MAS NMR spectra of the alkali-free samples (Fig. 6) are all rather similar, showing resonances only from the Q^1^, Q^2^(1Al(IV)), Q^2^_b_, and Q^2^_p_ sites (approx. − 78 to − 87 ppm) of the alumino-silicate chains in the C–A–S–H structure [55] and thereby confirming the basic structure of the C–A–S–H samples. Only minor differences between the spectra of the samples after 3 months and 2 years of curing are noticed for the different Al/Si ratios, suggesting that the basic silicate structure only experience minor modifications or refinements with prolonged curing time. Simulations of the ^29^Si MAS NMR spectra for the samples with Al/Si = 0.01 and 0.2 after curing for 2 years (not shown), using the four types of Si sites mentioned above, give average alumino silicate lengths of CL = 22.7 and 23.1, respectively, which are in according with the presence of long alumino silicate chains for C–A–S–H phases with low Ca/Si ratio [24, 36]. Moreover, for the Al/Si = 0.2 sample, this simulation gives A(IV)/Si = 0.116 ± 0.015, which is consistent with the value (0.106) derived from ^27^Al NMR (Table).

### C-A-S–H with 1 M NaOH

#### The effect of Al concentration on secondary phases

**Fig. 7:**
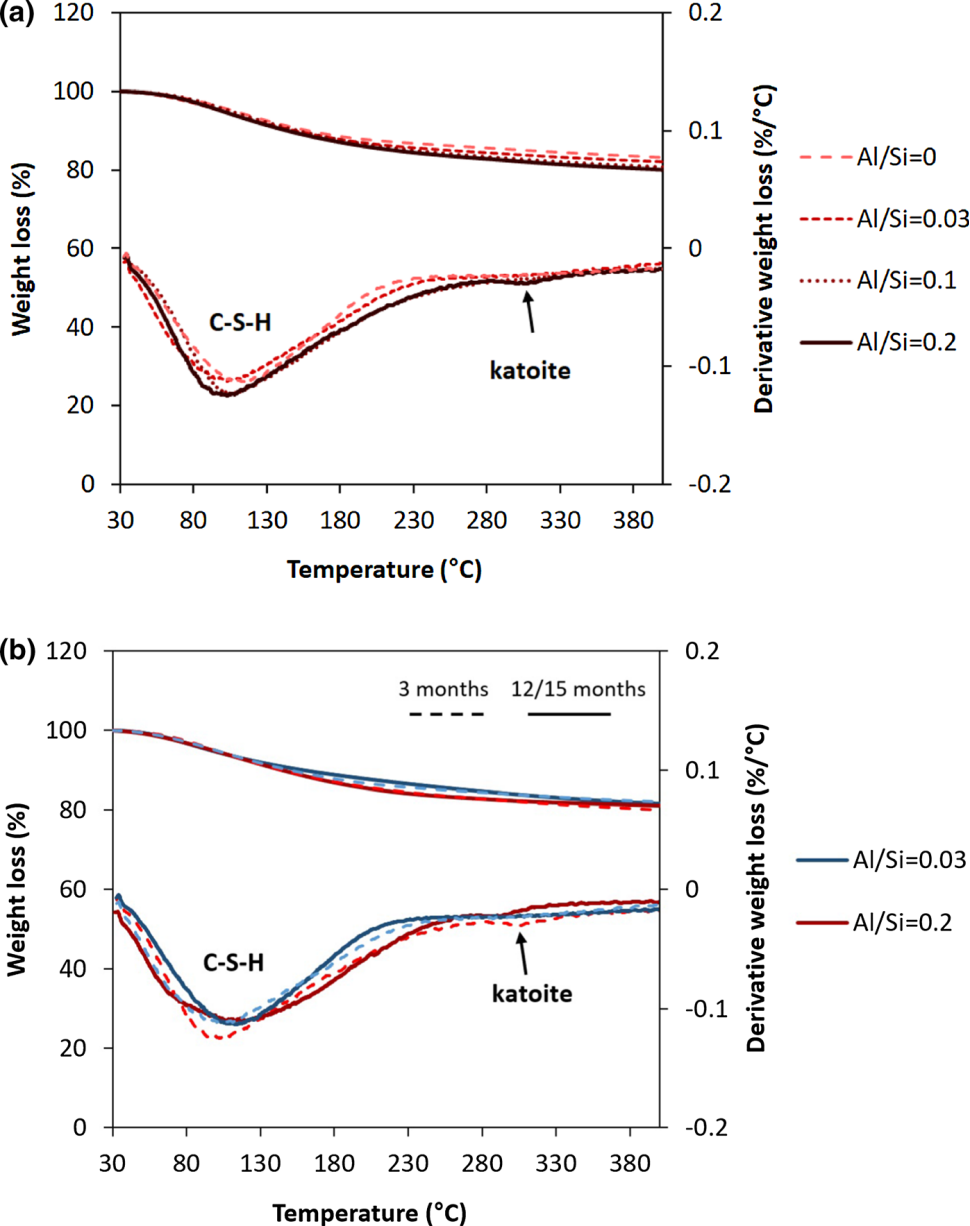
The effect of** a** Al content after 3 months equilibration and** b** equilibration time on secondary phases’ content in the presence of 1 M NaOH for target Ca/Si = 0.8. (The samples at target Al/Si = 0.2 were analyzed after 15 months instead of 12 months)

Figure 7 shows that also in the presence of 1 M NaOH katoite is present at target Al/Si ratio of 0.2, but absent at lower Al/Si, indicating the presence of less secondary phases at higher pH values, which is consistent with observations of [14] in KOH and of [55] in NaOH containing samples. The quantity of katoite present at target Al/Si ratio of 0.2 decreases with time also in 1 M NaOH as shown in Fig. 6b, while at target Al/Si of 0.1 and below again very little or no secondary phases are observed, in agreement with Al NMR measurements of these samples reported in [55].

**Fig. 8:**
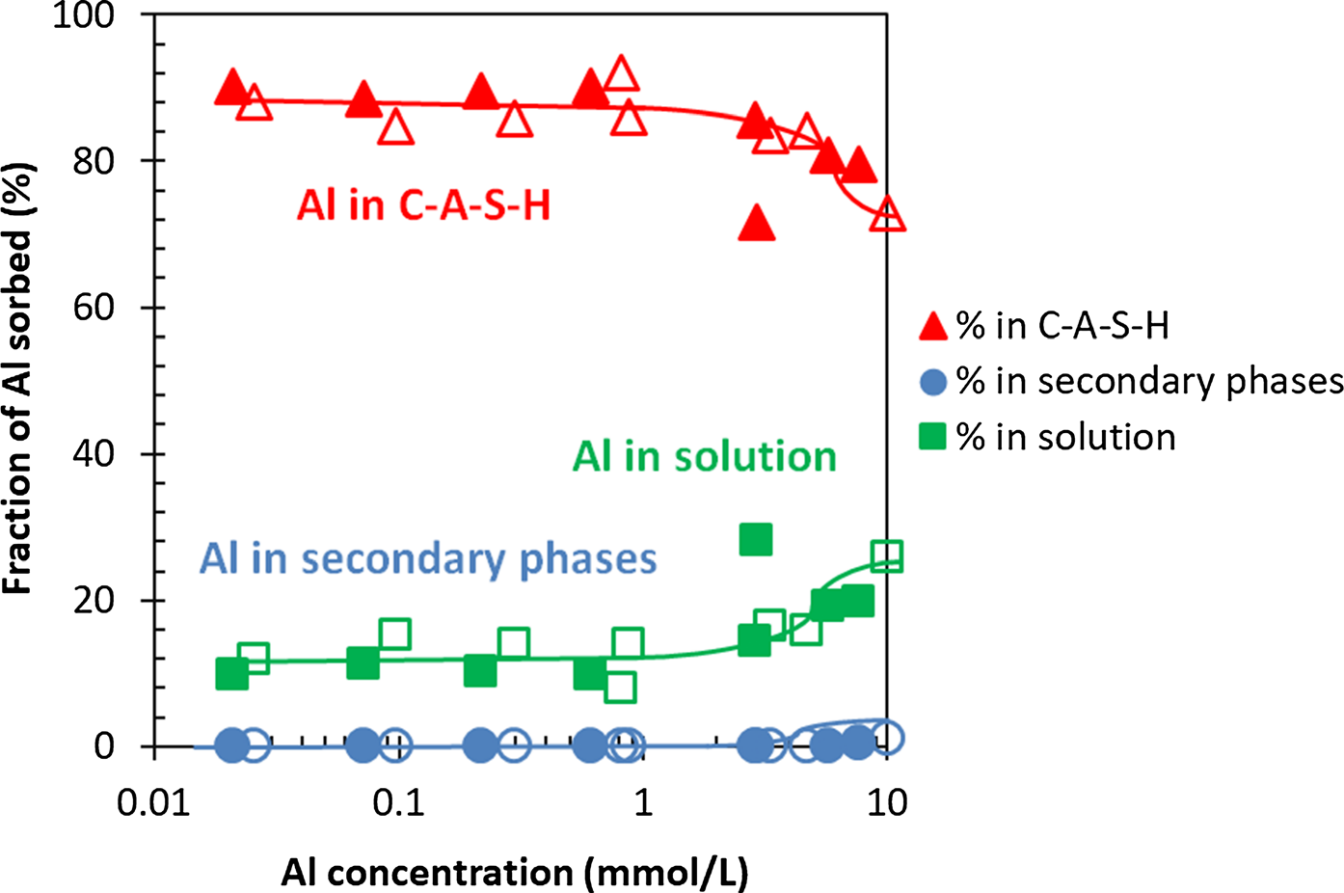
The Al fraction in solution, C–A–S–H and secondary phases vs. measured Al concentration for target Ca/Si = 0.8 in the presence of 1 M NaOH after 3 months (empty symbols) and 15 months (filled symbols) equilibration. (The lines serve as eye-guides only and the errors are smaller than the symbols' size)

Figure 8 illustrates again that roughly 90% of the total Al is bound in C–A–S–H at concentrations below 1 mmol/L, while secondary phases are absent. At high target Al/Si ratios (0.1, 0.15 and 0.2), an increase of the Al concentrations in solution is observed due to the high NaOH concentrations and secondary phases (katoite) present with a maximum content of 1.1% after 3 months (3% based on Al NMR, see [55]) and of 0.5% after 15 months equilibration as shown in Appendix G. Comparing Fig. 4 with Fig. 8 shows that the fraction of Al taken up in C–A–S–H depends not only on the quantity of Al containing secondary phases but on the Al concentrations in solution. At target Al/Si = 0.2, 26% of the total Al is in solution after 3 months in 1 M NaOH which decrease to 20% after 15 months. In contrast, only 0.1% after 3 months and < 0.01% after 12 months of the total Al is present in solution in the absence of NaOH. At high NaOH concentrations the content of secondary phases is much lower compared to no alkali samples, but the Al concentrations are much higher. Therefore, the content of secondary phases is not the only factor affecting the Al uptake in C–A–S–H but also the pH values and Al concentration in solution play a significant role.

#### The effect of Al concentration on C–A–S–H structure

**Fig. 9:**
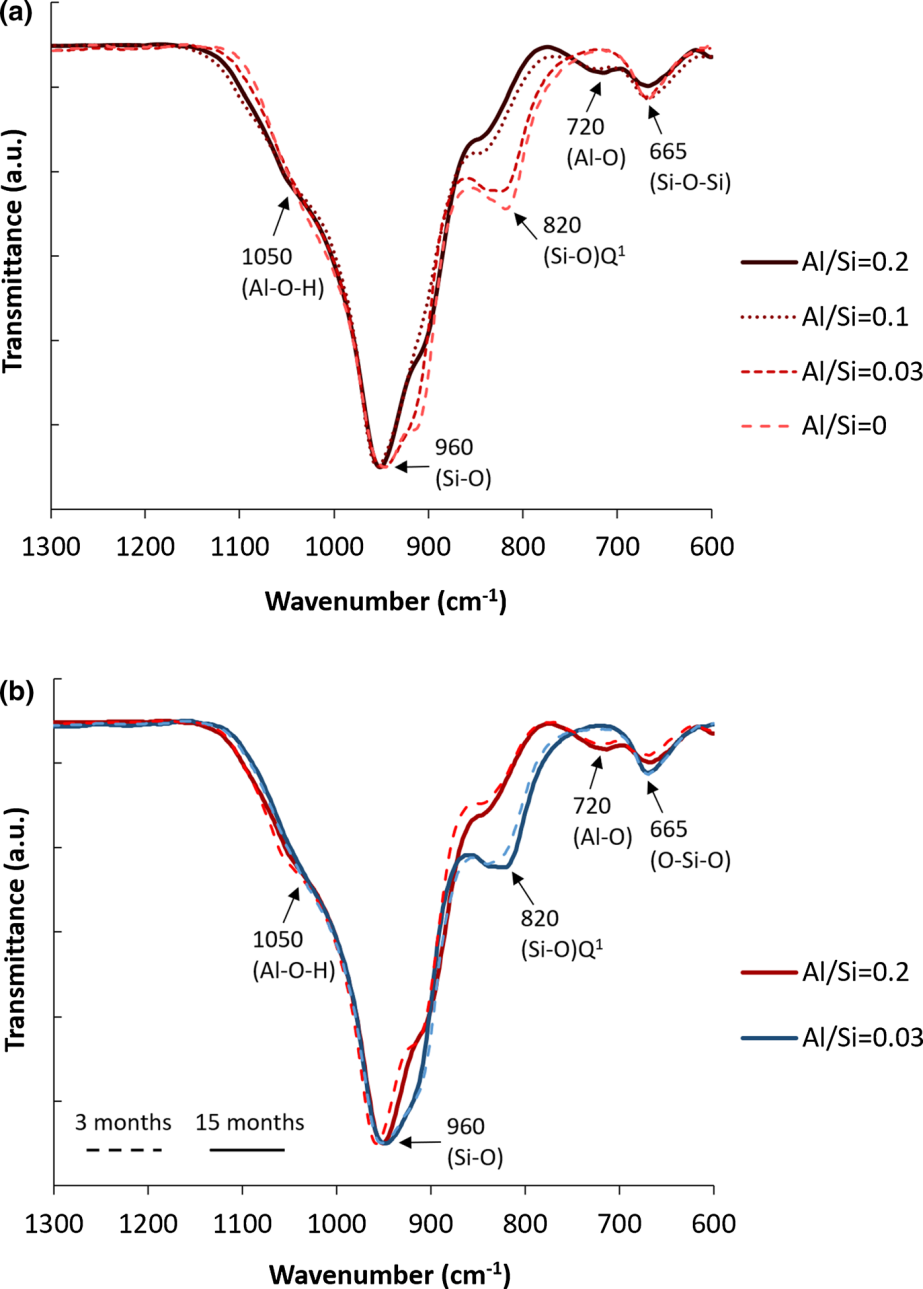
The FTIR spectra for C–A–S–H samples at target Ca/Si = 0.8 and in the presence of 1 M NaOH for** a** 15 months equilibration with different Al/Si ratios and** b** different equilibration times with target Al/Si ratios of 0.03 and 0.2

The FTIR spectra of C–A–S–H samples containing 1 M NaOH are shown in Fig. 9 (a) for 15 months equilibration with target Al/Si ratios from 0 to 0.2 and (b) different equilibration times with target Al/Si ratios of 0.03 and 0.2. Again, the intensity of the band at 665 cm^−1^ (Si–O–Si bending vibrations) drops with increasing Al content. Furthermore, an additional signal around 720 cm^−1^ appears at high target Al/Si ratios of 0.1 and 0.2, which is assigned to Al–O stretching vibrations of octahedrally coordinated Al [56], as present in secondary phases such as strätlingite, katoite and Al(OH)_3_. Figure 9 shows this band is absent in the absence of Al and in the presence of little Al (target Al/Si = 0.03). Moreover, the intensity of (Si–O) Q^1^ peak at 820 cm^−1^ decreases significantly with an increase in Al content, which indicates that the fraction of Si in Q^1^ sites is lowered as Al occupies previously empty bridging sites.

Figure 9b illustrates that the intensity of the shoulder at 1050 cm^−1^ (Al–O–H) does not significantly change with time, in contrast to the samples without alkali (Fig. 5b), indicating that the change with time are larger in alkali free samples and/or in samples with a low alkali content; in agreement with the only slightly decreasing Al concentrations over time in the presence of 0.5 M and 1 M NaOH reported in [38]. The peaks at 920 cm^−1^ and 960 cm^−1^ (Si–O stretching vibrations) move to a lower wavenumber from 3 to 15 months.

### C–A–S–H with different NaOH concentrations

#### The effect of NaOH concentration on Al sorption isotherm

**Fig. 10:**
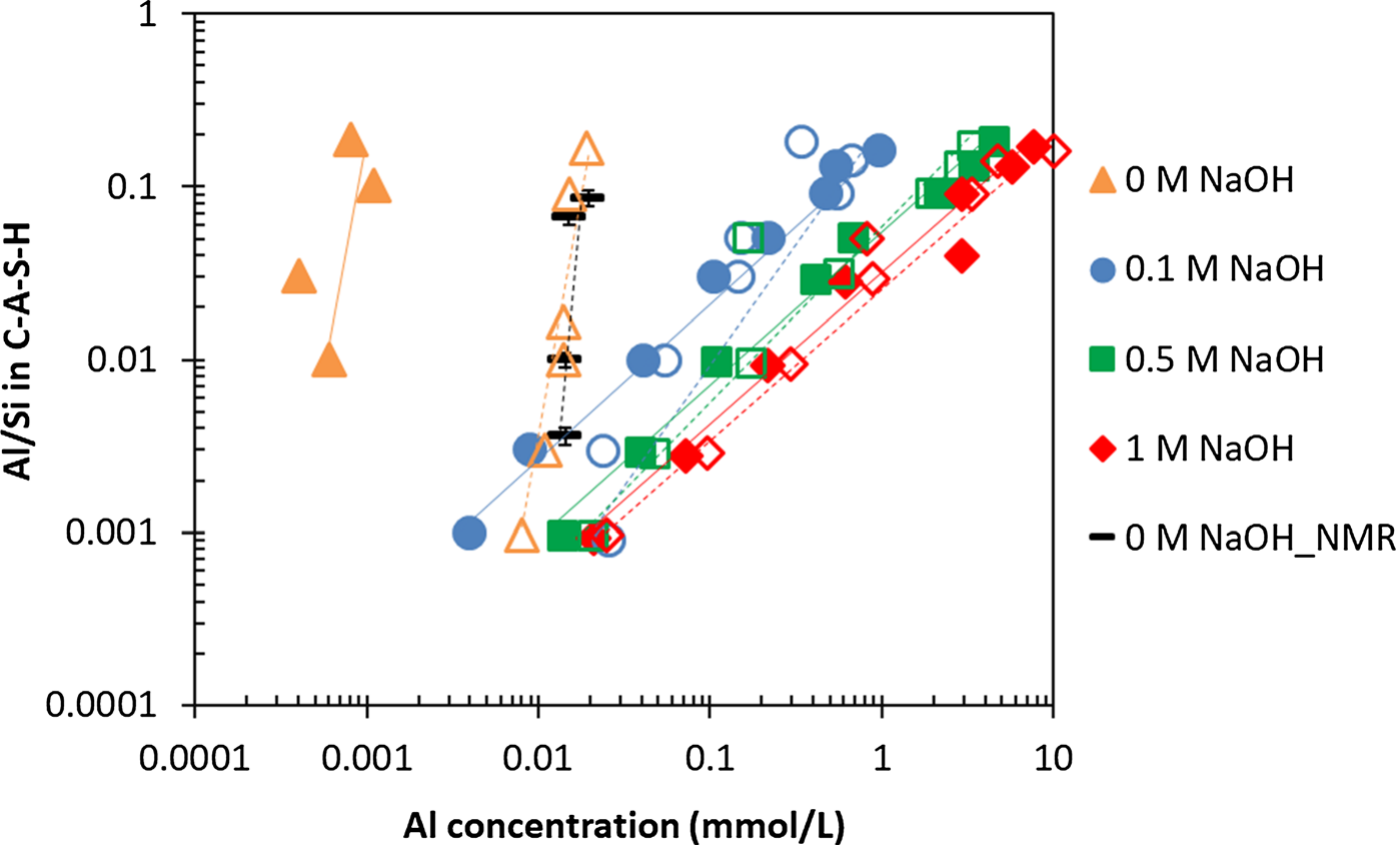
The Al sorption isotherm on C–A–S–H for target Ca/Si = 0.8 recorded after different equilibration times. The 3 months samples are represented by empty symbols and one year samples indicated by filled symbols. The alkali-free samples analyzed with.^27^Al MAS NMR after 3 months equilibration are indicated with black symbols. The samples at target Al/Si ≥ 0.05 were analyzed after 15 months instead of 1 year. The lines indicate the slope of the increase; slopes ≤ 1 indicate sorption; slopes > 1 indicate precipitation of an additional solid. (The errors for mass-balance calculations are smaller than the symbols' size)

The effect of NaOH concentrations on Al sorption by C–A–S–H after 3 months and 1 year is shown in Fig. 10 as Al sorption isotherm. Higher Al concentrations increase the Al uptake in C–S–H. This agrees with other experimental studies on Al sorption in C–S–H at relatively high Al content (Al/Si ≥ 0.05) [14, 16, 30, 34], as well as at low Al contents (Al/Si from 0.001 to 0.03) [35, 38]. At low Al/Si ratios (≤ 0.03), the Al uptake in C–S–H increases from 3 months to 1 year. This increase is much more significant at 0 and 0.1 M than at high NaOH concentrations (0.5 and 1 M). However, at high Al/Si ratios (≥ 0.05) an increase in the Al uptake in C–S–H is only observed in samples without alkali or with little (0.1 M) NaOH content. In the presence of 0.5 and 1 M NaOH, no obvious increase for Al uptake in C-S–H is observed. However, as Al concentrations are higher at high Al/Si ratios, small concentrations changes over time may be difficult to observe.

The large range studied here confirms a linear relationship between Al in C–A–S–H and Al in solution over more than 2 orders of magnitude. The linear trend demonstrates an Al uptake on one or various types of sorption sites, with a relative high capacity of up to Al/Si ≥ 0.2 at target Ca/Si = 0.8. The continuous uptake and relative high capacity are consistent with an uptake of Al in the bridging sites of the silica chains as suggested by NMR studies (Figs. 2, 6, [20, 32, 55]). A slope of ≈ 1 is observed between the logarithm of the Al concentration and Al in C–S–H in the presence of NaOH, while a slope of ≈ 2 to 4 is present in the alkali free C–A–S–H, which could point towards the formation of an unidentified surface precipitate or an additional secondary phase even at those very low aluminum concentrations. Note that those solutions are clearly undersaturated with respect to Al containing hydrates such as Al(OH)_3_, strätlingite and katoite (see Appendix C), although no secondary phases are present at target Al/Si ≤ 0.01. Thus, either surface precipitate containing tetrahedrally coordinated Al or a zeolitic precursor (with ^27^Al NMR signals typically at around 50 to 60 ppm) might have formed in low quantity [74, 75] since the alkali-free solutions are strongly oversaturated with respect to chabazite and Ca-gismondine as indicated in Appendix C. Note that neither XRD nor NMR indicate the presence of any crystalline phases [38]. Moreover chabazite or any other zeolitic precursor present in low quantity will not be visible neither by TGA (as their main weight loss will occur below 200 °C, i.e. in the range of the C–A–S–H signals [47]) nor by FTIR, where their main signals (between ~ 900 cm^−1^ and 1000 cm^−1^) are in the same range as the C–A–S–H main signals.

**Fig. 11:**
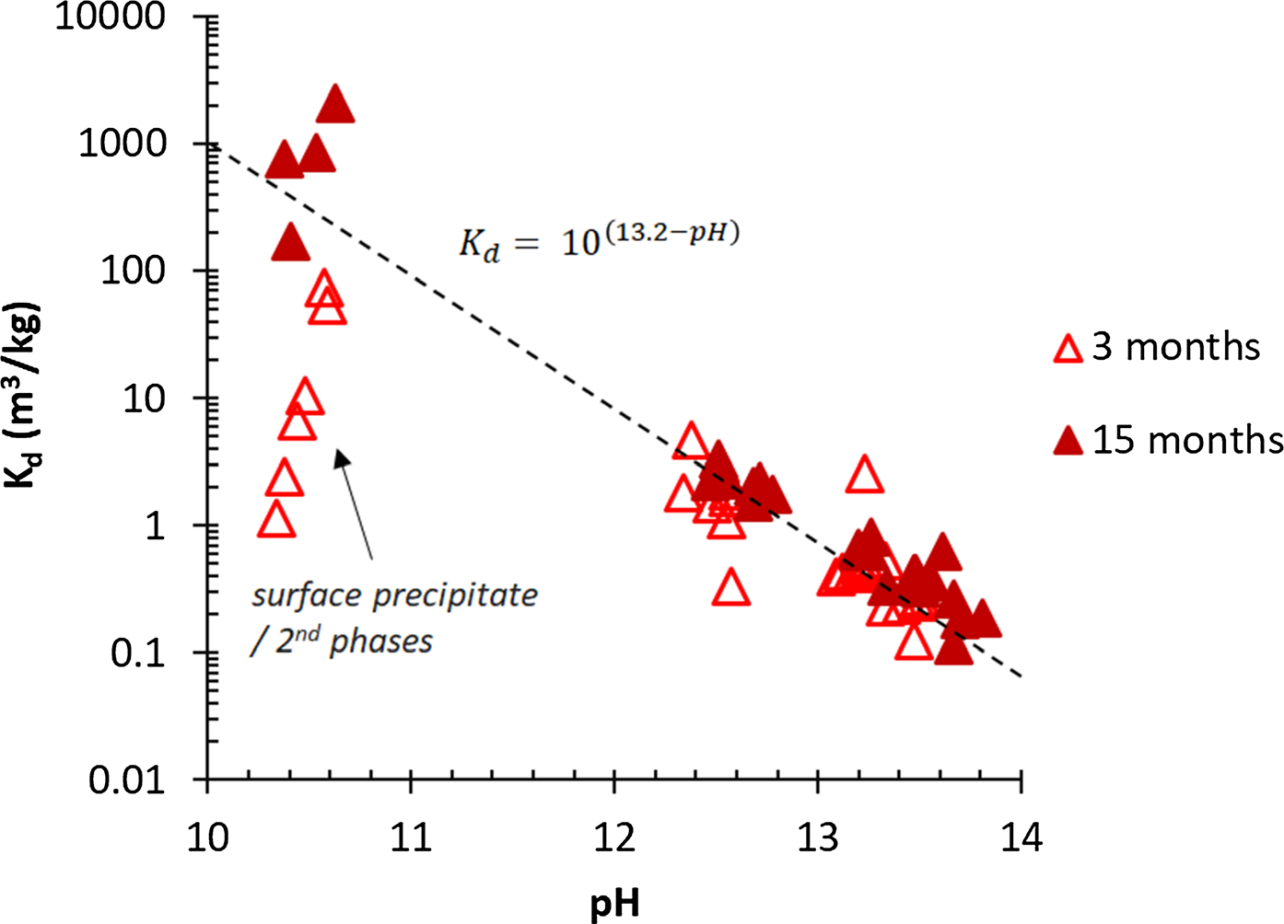
The pH dependence of Al sorption on C–A–S–H for target Ca/Si = 0.8. The *K*_*d*_ can be expressed as 10.^(13.2−pH)^ as visualized by the dashed line. (The errors are smaller than the symbols’ size)

The uptake of Al into C-S–H phases can also be imitated using a *K*_*d*_ value, which is the distribution coefficient and describes the ratio of the quantity of Al adsorbed to the quantity of the Al remaining in the solution. The *K*_*d*_ values for different Al/Si ratios were calculated according to Eq. 3 and plotted versus pH values in Fig. 11. The total amount of bound Al decreases as is visible in the lowering of the *K*_*d*_ values from ≈ 600 m^3^/kg (without NaOH) after 1 year to ≈ 0.2 m^3^/kg in 1 M NaOH. The decrease of Al uptake by C-S–H with increasing the pH value (Fig. 11) is comparable to the decrease of Fe(III) uptake by titanium dioxide (TiO_2_) with increasing the pH value [76], which is present mainly as negatively charged hydroxide complex (Fe(OH)_4_^−^) at pH values above 10. The aqueous aluminum speciation depends on pH; negatively charged Al(OH)_4_^−^ dominates at pH > 7 as explained in [35, 77]. At higher pH values the fraction of the Al(OH)_4_^−^ species in solution increases, which decreases the tendency of Al to sorb on solids such as C–S–H [35], such that the *K*_*d*_ values decrease with increasing the pH value. After 1 year, the 1:1 decrease of the *K*_*d*_ values with pH confirms the important role of the solution speciation on the Al binding in C–S–H. In the absence of NaOH, the Al uptake is strongly influenced by the presence of secondary phases as discussed above, resulting in scattered *K*_*d*_ values.

The *K*_*d*_ values of ≈ 600 m^3^/kg for Al in the absence of alkali are comparable to the *K*_*d*_ values of ≈ 700 m^3^/kg for Fe(III) on C–S–H reported in [76]. However, they are considerably higher than the *K*_*d*_ values in the range of 0.1 m^3^/kg to 6 m^3^/kg observed for bivalent cations such as Fe(II), Ba(II) or Sr(II) [78–80].

#### The effect of NaOH concentration on secondary phases

**Fig. 12:**
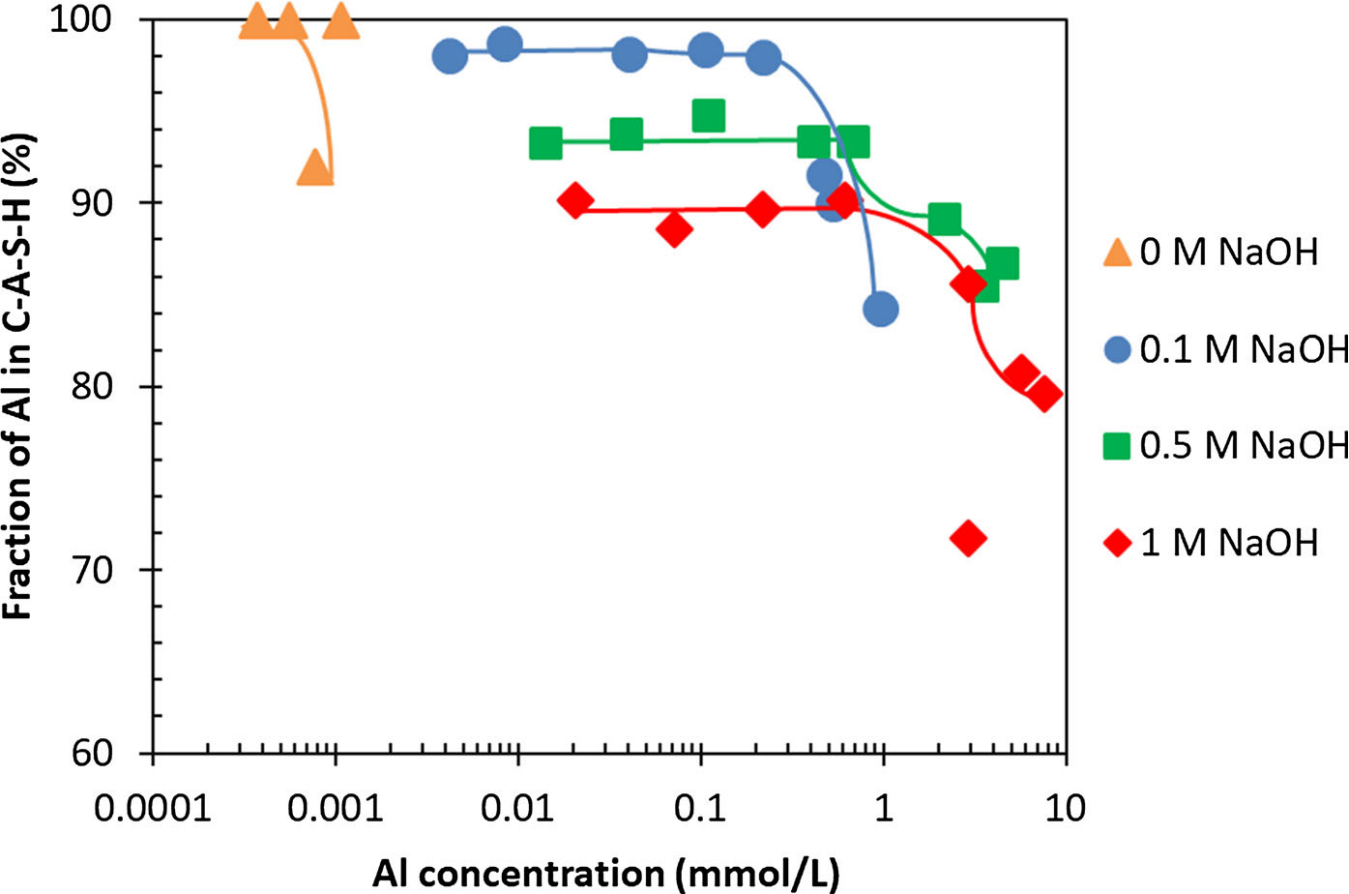
The Al fraction in C–A–S–H for target Ca/Si = 0.8 in the absence of NaOH and presence of 0.1, 0.5 and 1 M NaOH after 1 year equilibration. Samples at target Al/Si ≥ 0.05 were analyzed after 15 months instead of 1 year. (The errors are smaller than the symbols’ size)

**Fig. 13:**
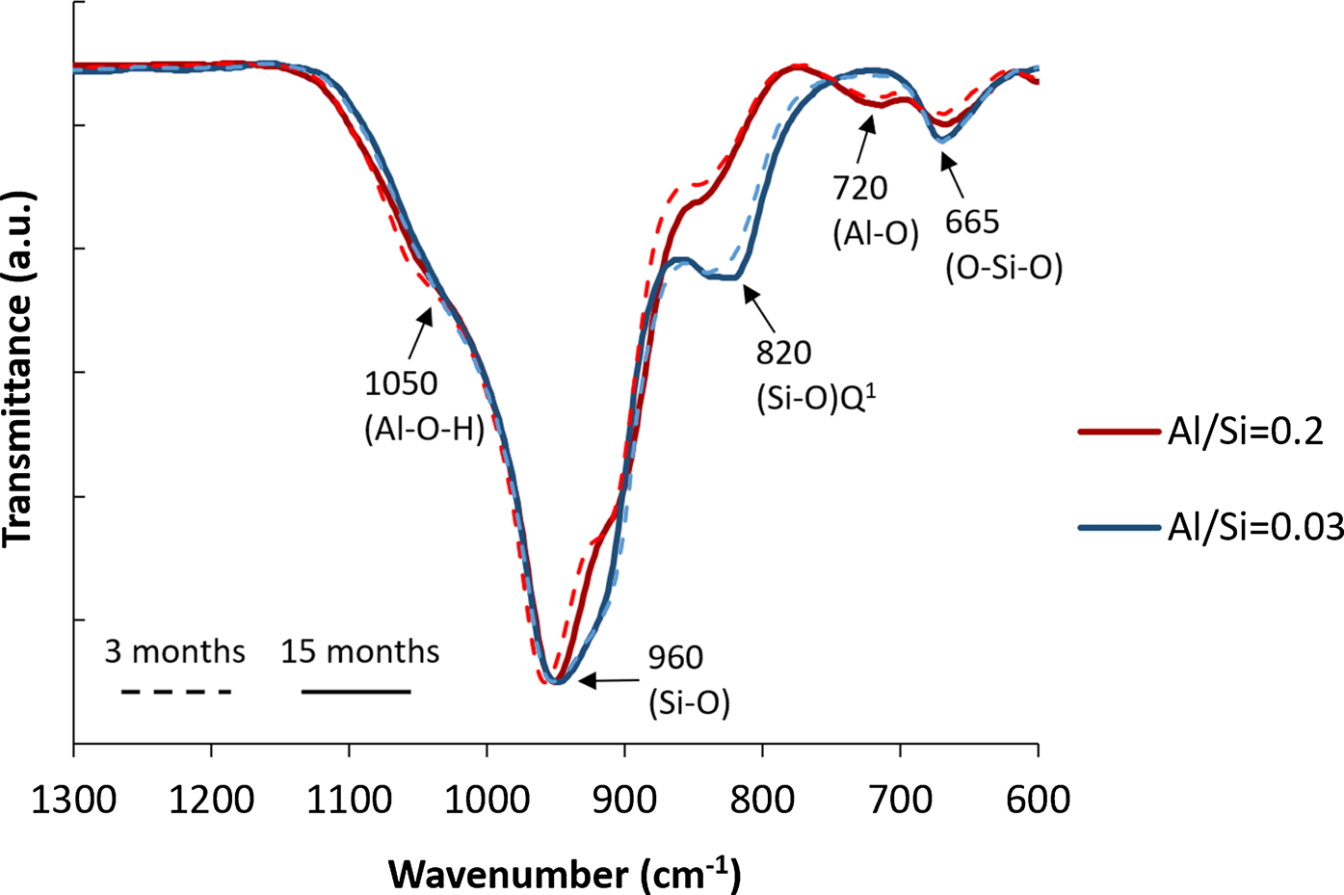
The FTIR spectra for C–A–S–H samples in the absence of NaOH and presence of 1 M NaOH for target Ca/Si = 0.8 with target Al/Si ratios of 0.03 and 0.2 after 3 months equilibration. Dashed lines with light colors represent the samples without NaOH and solid lines with dark colors show those with 1 M NaOH

The sorbed Al fraction in C–A–S–H for different NaOH concentrations after 1 year equilibration is shown in Fig. More Al is present in C–A–S–H at low NaOH concentrations in agreement with the higher *K*_*d*_ values at low pH values. At all NaOH concentrations, the fraction of Al bound in C–A–S–H decreases with an increase in Al concentrations due to the presence of secondary phases. High alkali concentrations lower the amount of secondary phases as shown in [35], leading to less secondary phases at lower Al concentrations and thus at intermediate Al concentrations to a higher bounding of Al in C–A–S–H compared to samples without any alkalis. The Al fraction in C–A–S–H for 3 months equilibration is shown in Appendix E. The effect of NaOH concentrations on the content of secondary phases is presented in Appendix G (Fig. 12 and 13).

#### The effect of NaOH concentration on C–A–S–H structure

Fig. shows the structure of C–A–S–H with target Al/Si ratios of 0.03 and 0.2 after 3 months equilibration for different NaOH concentrations. Comparing the FTIR spectra in dashed lines (alkali-free) with full lines (1 M NaOH), it becomes clear that the intensity of Q^1^ sites at 820 cm^−1^ is higher in a 1 M NaOH solution than in the absence of NaOH. This indicates a shorter silica chain length in samples containing more NaOH, which agrees ^29^Si MAS NMR observations [14, 29, 81, 82]. At both target Al/Si ratios of 0.03 and 0.2, the intensity of Si–O stretching vibration at 920 cm^−1^ increases significantly with increasing the NaOH concentrations. The bands at 920 cm^−1^ and 960 cm^−1^ move to a shorter wavenumber at higher NaOH concentrations, indicating depolymerization of the silica chains [21]. Furthermore, increasing the NaOH concentration leads to an increase in the intensity of the shoulder at 1050 cm^−1^. Moreover, the signal at 720 cm^−1^ for Al–O stretching vibrations of octahedrally coordinated Al appears only in the presence of 1 M NaOH, which indicates that the amount of secondary, as well as the C–A–S–H structure and the Al uptake varies with the NaOH concentrations for low Ca/Si C–A–S–H phases. Therefore, different NaOH concentrations not only change the content of secondary phases and the Al concentrations in solution, but also affect the structure of C–A–S–H.

## Conclusions

The effect of aluminum concentration on Al uptake in low Ca/Si C–S–H (Ca/Si = 0.8) was investigated using sorption isotherm experiments over a wide range of target Al/Si ratio from 0.001 to 0.2. The Al uptake in C–S–H was observed by FTIR and NMR spectroscopy, where the intensity of signals assigned to Al-O bands in C–A–S–H structure increased with an increase in Al content. At low Al/Si ratios, Al was exclusively bound in C–A–S–H, while at high Al/Si ratios secondary phases containing Al such as katoite, strätlingite, and Al(OH)_3_ were formed in addition to the C–A–S–H phase, lowering the fraction of Al in C–A–S–H. In the absence of alkali hydroxide, secondary phases were observed by TGA and Al NMR at target Al/Si ≥ 0.03, while the sorption isotherms indicated the presence of traces of secondary phases even at lower Al/Si ratios.

Al sorption isotherms showed more Al in C–S–H from 3 months to 1 year, in particular in the absence of NaOH and at low Al concentrations than at high Al concentrations where secondary phases were present. This indicated a slow rearrangement of the C–A–S–H phases with time which increases also the Al incorporation in C–S–H. The initially low uptake might also be related to the experimental procedure used, which favors the initial formation of Al containing secondary phases leading to low Al concentrations. Over time, less Al was bound in secondary phases and more Al was bound in C–A–S–H.

The presence of NaOH progressively shifted the precipitation of secondary phases to higher Al/Si ratios; to target Al/Si ≥ 0.1 at 0.1 M NaOH and to target Al/Si ≥ 0.2 at 1 M NaOH. The absence of secondary phases in the presence of NaOH led to a higher fraction of Al bound in C–A–S–H at high Al/Si ratios. At very low Al concentrations, however, the high pH values lowered Al uptake in C–S–H as Al(OH)_4_^−^ has a strong tendency to remain in solution. FTIR spectra suggested a shortening in the silica chain length with increasing NaOH concentrations, independent of its Al content.

The Al sorption isotherm show a linear increase of the amount of Al in C–A–S–H with Al concentration in solution. The linear trend suggested an Al uptake on one or various types of sorption sites, with a high sorption capacity. This information is consistent with the Al uptake in the bridging position of the silica chains as proposed by NMR studies [20, 32]. The steep increase of Al uptake in C–S–H in the absence of NaOH tentatively indicated the formation of a surface precipitate or of a not clearly identified secondary phase.

The decrease of the distribution coefficients, *K*_*d*_ values, of Al on C–S–H from ≈ 600 m^3^/kg in the absence of NaOH to ≈ 0.2 m^3^/kg in the presence of 1 M NaOH indicated a 1:1 decrease of Al uptake by C–S–H with increasing the pH values.

## Supplementary Information

Below is the link to the electronic supplementary material.

Supplementary file1 (DOCX 283 KB)
