# Supplementary material for: A long-term study on structural changes in calcium aluminate silicate hydrates
Source: Mater Struct. 2022 Nov 24;55(10):243. doi: 10.1617/s11527-022-02080-x (PMC9700620; doi:10.1617/s11527-022-02080-x)

**Supporting information for**

**A Long-term Study on Structural Changes in Calcium Aluminate Silicate Hydrates**

Sonya Barzgar  ^a,b+*^, Yiru Yan ^a^, Mohamed Tarik ^c^, Jorgen Skibsted ^d^, Christian Ludwig ^b,c^, Barbara Lothenbach ^a,e^

^a^ Empa, Concrete & Asphalt Laboratory, CH-8610 Dübendorf, Switzerland

^b^ École Polytechnique Fédéral de Lausanne (EPFL), **ENAC IIE GR-LUD,** CH-1015 Lausanne, Switzerland

^c^ Paul Scherrer Institute (PSI), ENE LBK CPM, 5232 Villigen PSI, Switzerland

^d^ Department of Chemistry and Interdisciplinary Nanoscience Center, Aarhus University, Langelandsgade 140, DK-8000 Aarhus C, Denmark

^e^ NTNU, Department of Structural Engineering, Trondheim, Norway

**Corresponding Author**: Sonya Barzgar; [sonya.barzgar@alumni.epfl.ch](mailto:sonya.barzgar@alumni.epfl.ch)

**^+^Present/Permanent address:** National Physical Laboratory (NPL), Department of Emissions and Atmospheric Methrology, Hampton Road, TW11 0LW Teddington, United Kingdom

Journal name: This article has been submitted to the Materials and Structures.

Appendix A. The solid phase composition of C-A-S-H. The Ca/Si and Al/Si ratios were calculated from mass-balance.

| Target Ca/Si | Target Al/Si | NaOH (M) | Time (months) | strätlingite (wt%) | Al(OH)_3_ (wt%) | Katoite  (wt%) | CaCO_3_ (wt%) | Ca/Si in  C-A-S-H | Al/Si in  C-A-S-H | Na/Si  in solid | H_2_O/Si in solid |
| --- | --- | --- | --- | --- | --- | --- | --- | --- | --- | --- | --- |
| 0.8 | 0 | 0 | 3 | n.m. | n.m. | n.m. | n.m. | 0.81 | 0 | 0 | 1.18 |
| 0.8 | 0.001 | 0 | 3 | n.m. | n.m. | n.m. | n.m. | 0.80 | 0.0010* | 0 | 1.18 |
| 0.8 | 0.003 | 0 | 3 | n.m. | n.m. | n.m. | n.m. | 0.81 | 0.0030* | 0 | 1.18 |
| 0.8 | 0.01 | 0 | 3 | n.m. | n.m. | n.m. | n.m. | 0.80 | 0.0100 | 0 | 1.19 |
| 0.8 | 0.03 | 0 | 3 | n.o. | 0.29 | 1.7 | n.o. | 0.79 | 0.0160 | 0 | 1.20 |
| 0.8 | 0.1 | 0 | 3 | n.o. | 0.14 | 1.4 | n.o. | 0.80 | 0.0911 | 0 | 1.24 |
| 0.8 | 0.2 | 0 | 3 | n.o. | 1.4 | 2.1 | n.o. | 0.79 | 0.1686 | 0 | 1.30 |
| 0.8 | 0 | 0 | 12 | n.m. | n.m. | n.m. | n.m. | 0.80 | 0 | 0 | 1.18 |
| 0.8 | 0.001 | 0 | 12 | n.m. | n.m. | n.m. | n.m. | 0.80 | 0.0010* | 0 | 1.18 |
| 0.8 | 0.003 | 0 | 12 | n.m. | n.m. | n.m. | n.m. | 0.80 | 0.0030* | 0 | 1.18 |
| 0.8 | 0.01 | 0 | 12 | n.m. | n.m. | n.m. | n.m. | 0.80 | 0.0100 | 0 | 1.18 |
| 0.8 | 0.03 | 0 | 12 | n.o. | n.o. | n.o. | n.o. | 0.80 | 0.0300 | 0 | 1.19 |
| 0.8 | 0.1 | 0 | 12 | n.o. | n.o. | n.o. | n.o. | 0.80 | 0.1008 | 0 | 1.23 |
| 0.8 | 0.2 | 0 | 12 | 0.58 | n.o. | 2.1 | n.o. | 0.78 | 0.1864 | 0 | 1.42 |
| 0.8 | 0 | 0.1 | 3 | n.m. | n.m. | n.m. | n.m. | 0.81 | 0 | 0.19 | 1.18 |
| 0.8 | 0.001 | 0.1 | 3 | n.m. | n.m. | n.m. | n.m. | 0.81 | 0.0009 | 0.14 | 1.18 |
| 0.8 | 0.003 | 0.1 | 3 | n.m. | n.m. | n.m. | n.m. | 0.81 | 0.0029 | 0.19 | 1.18 |
| 0.8 | 0.01 | 0.1 | 3 | n.m. | n.m. | n.m. | n.m. | 0.81 | 0.0098 | 0.19 | 1.18 |
| 0.8 | 0.03 | 0.1 | 3 | n.o. | n.o. | n.o. | 1.2 | 0.79 | 0.0295 | 0.17 | 1.19 |
| 0.8 | 0.05 | 0.1 | 3 | n.o. | n.o. | n.o. | 1.4 | 0.82 | 0.051 | n.m. | 1.15 |
| 0.8 | 0.1 | 0.1 | 3 | n.o. | n.o. | 0.88 | 1.5 | 0.79 | 0.094 | n.m. | 1.15 |
| 0.8 | 0.15 | 0.1 | 3 | n.o. | n.o. | 1.1 | 1.6 | 0.79 | 0.145 | n.m. | 1.19 |
| 0.8 | 0.2 | 0.1 | 3 | 0.58 | n.o. | 2.1 | 0.9 | 0.81 | 0.192 | n.m. | 1.24 |
| 0.8 | 0 | 0.1 | 12 | n.m. | n.m. | n.m. | n.m. | 0.81 | 0 | 0.20 | 0.94 |
| 0.8 | 0.001 | 0.1 | 12 | n.m. | n.m. | n.m. | n.m. | 0.81 | 0.0010 | 0.20 | 0.94 |
| 0.8 | 0.003 | 0.1 | 12 | n.m. | n.m. | n.m. | n.m. | 0.81 | 0.0030 | 0.20 | 0.94 |
| 0.8 | 0.01 | 0.1 | 12 | n.m. | n.m. | n.m. | n.m. | 0.80 | 0.0099 | 0.19 | 0.94 |
| 0.8 | 0.03 | 0.1 | 12 | n.o. | n.o. | n.o. | n.o. | 0.81 | 0.0297 | 0.19 | 0.95 |
| 0.8 | 0.05 | 0.1 | 15 | n.o. | n.o. | n.o. | 0.4 | 0.80 | 0.049 | n.m. | 0.77 |
| 0.8 | 0.1 | 0.1 | 15 | n.o. | n.o. | 1.1 | 0.7 | 0.79 | 0.093 | n.m. | 0.96 |
| 0.8 | 0.15 | 0.1 | 15 | n.o. | n.o. | 2.1 | 1.1 | 0.78 | 0.139 | n.m. | 1.25 |
| 0.8 | 0.2 | 0.1 | 15 | 1.74 | n.o. | 2.8 | 0.9 | 0.78 | 0.174 | n.m. | 1.28 |
| 0.8 | 0 | 0.5 | 3 | n.m. | n.m. | n.m. | n.m. | 0.84 | 0 | 0.25 | 1.04 |
| 0.8 | 0.001 | 0.5 | 3 | n.m. | n.m. | n.m. | n.m. | 0.84 | 0.0010 | 0.20 | 1.05 |
| 0.8 | 0.003 | 0.5 | 3 | n.m. | n.m. | n.m. | n.m. | 0.82 | 0.0028 | 0.19 | 1.02 |
| 0.8 | 0.01 | 0.5 | 3 | n.m. | n.m. | n.m. | n.m. | 0.82 | 0.0095 | 0.19 | 1.03 |
| 0.8 | 0.03 | 0.5 | 3 | n.o. | n.o. | n.o. | 3.7 | 0.86 | 0.0312 | 0.11 | 1.15 |
| 0.8 | 0.05 | 0.5 | 3 | n.o. | n.o. | 0.35 | 2.5 | 0.78 | 0.048 | n.m. | 1.07 |
| 0.8 | 0.1 | 0.5 | 3 | n.o. | n.o. | n.o. | 2.3 | 0.79 | 0.092 | n.m. | 1.10 |
| 0.8 | 0.15 | 0.5 | 3 | n.o. | n.o. | 1.3 | 1.7 | 0.83 | 0.138 | n.m. | 1.19 |
| 0.8 | 0.2 | 0.5 | 3 | n.o. | n.o. | 1.8 | 0.9 | 0.80 | 0.179 | n.m. | 1.18 |
| 0.8 | 0 | 0.5 | 12 | n.m. | n.m. | n.m. | n.m. | 0.81 | 0 | 0.48 | 0.95 |
| 0.8 | 0.001 | 0.5 | 12 | n.m. | n.m. | n.m. | n.m. | 0.81 | 0.0009 | 0.33 | 0.94 |
| 0.8 | 0.003 | 0.5 | 12 | n.m. | n.m. | n.m. | n.m. | 0.82 | 0.0029 | 0.39 | 0.96 |
| 0.8 | 0.01 | 0.5 | 12 | n.m. | n.m. | n.m. | n.m. | 0.81 | 0.0097 | 0.24 | 0.96 |
| 0.8 | 0.03 | 0.5 | 12 | n.o. | n.o. | n.o. | n.o. | 0.81 | 0.0286 | 0.24 | 0.96 |
| 0.8 | 0.05 | 0.5 | 15 | n.o. | n.o. | n.o. | n.o. | 0.81 | 0.047 | n.m. | 0.91 |
| 0.8 | 0.1 | 0.5 | 15 | n.o. | n.o. | n.o. | 0.7 | 0.80 | 0.090 | n.m. | 1.15 |
| 0.8 | 0.15 | 0.5 | 15 | n.o. | n.o. | 0.70 | n.o. | 0.84 | 0.136 | n.m. | 0.86 |
| 0.8 | 0.2 | 0.5 | 15 | n.o. | n.o. | 0.49 | n.o. | 0.83 | 0.181 | n.m. | 0.94 |
| 0.8 | 0 | 1 | 3 | n.m. | n.m. | n.m. | n.m. | 0.87 | 0 | 0.15 | 1.14 |
| 0.8 | 0.001 | 1 | 3 | n.m. | n.m. | n.m. | n.m. | 0.88 | 0.0010 | -0.16 | 1.16 |
| 0.8 | 0.003 | 1 | 3 | n.m. | n.m. | n.m. | n.m. | 0.90 | 0.0029 | -0.27 | 1.18 |
| 0.8 | 0.01 | 1 | 3 | n.m. | n.m. | n.m. | n.m. | 0.87 | 0.0094 | 0.10 | 1.16 |
| 0.8 | 0.03 | 1 | 3 | n.o. | n.o. | n.o. | 2.0 | 0.87 | 0.0290 | 0.00 | 1.20 |
| 0.8 | 0.05 | 1 | 3 | n.o. | n.o. | n.o. | 2.6 | 0.81 | 0.048 | n.m. | 1.12 |
| 0.8 | 0.1 | 1 | 3 | n.o. | n.o. | n.o. | 2.8 | 0.79 | 0.086 | n.m. | 1.13 |
| 0.8 | 0.15 | 1 | 3 | n.o. | n.o. | n.o. | 3.0 | 0.82 | 0.135 | n.m. | 1.20 |
| 0.8 | 0.2 | 1 | 3 | n.o. | n.o. | 0.35 | 3.1 | 0.84 | 0.161 | n.m. | 1.33 |
| 0.8 | 0 | 1 | 12 | n.m. | n.m. | n.m. | n.m. | 0.83 | 0 | 0.39 | 0.91 |
| 0.8 | 0.001 | 1 | 12 | n.m. | n.m. | n.m. | n.m. | 0.82 | 0.0009 | 0.19 | 0.91 |
| 0.8 | 0.003 | 1 | 12 | n.m. | n.m. | n.m. | n.m. | 0.82 | 0.0028 | 0.44 | 0.91 |
| 0.8 | 0.01 | 1 | 12 | n.m. | n.m. | n.m. | n.m. | 0.83 | 0.0094 | 0.49 | 0.92 |
| 0.8 | 0.03 | 1 | 12 | n.o. | n.o. | n.o. | n.o. | 0.82 | 0.0281 | -0.10 | 0.92 |
| 0.8 | 0.05 | 1 | 15 | n.o. | n.o. | n.o. | n.o. | 0.84 | 0.037 | n.m. | 0.95 |
| 0.8 | 0.1 | 1 | 15 | n.o. | n.o. | n.o. | 0.8 | 0.83 | 0.089 | n.m. | 1.12 |
| 0.8 | 0.15 | 1 | 15 | n.o. | n.o. | n.o. | 0.6 | 0.83 | 0.128 | n.m. | 1.15 |
| 0.8 | 0.2 | 1 | 15 | n.o. | n.o. | 0.35 | 0.4 | 0.84 | 0.168 | n.m. | 1.25 |

(n.m.: not measured; n.o.: not observed; *: Al concentrations in the solution are below the DL of ICP-OES and Al/Si ratios calculated considering Al concentration equals to zero.)

Appendix B. Elemental concentrations in aqueous solution.

| Target Ca/Si | Target Al/Si | NaOH (M) | Time (months) | [Na] (mmol/L) | [Ca]  (mmol/L) | [Si]  (mmol/L) | [Al] (mmol/L) | [OH^-^] (mmol/L) | pH |
| --- | --- | --- | --- | --- | --- | --- | --- | --- | --- |
| 0.8 | 0 | 0 | 3 | n.m. | 0.53 | 2.23 | <0.0004 | 0.22 | 10.35 |
| 0.8 | 0.001 | 0 | 3 | n.m. | 0.82 | 2.13 | 0.008 | 0.22 | 10.34 |
| 0.8 | 0.003 | 0 | 3 | n.m. | 0.49 | 2.28 | 0.011 | 0.24 | 10.38 |
| 0.8 | 0.01 | 0 | 3 | n.m. | 1.08 | 2.68 | 0.014 | 0.28 | 10.44 |
| 0.8 | 0.03 | 0 | 3 | n.m. | 0.67 | 3.12 | 0.014 | 0.30 | 10.48 |
| 0.8 | 0.1 | 0 | 3 | n.m. | 0.50 | 3.02 | 0.015 | 0.39 | 10.59 |
| 0.8 | 0.2 | 0 | 3 | n.m. | 0.79 | 3.28 | 0.019 | 0.37 | 10.57 |
| 0.8 | 0 | 0 | 12 | n.m. | 1.34* | 2.03 | <0.0004 | 0.42 | 10.32 |
| 0.8 | 0.001 | 0 | 12 | n.m. | 1.06* | 1.77 | <0.0004 | 0.21 | 10.33 |
| 0.8 | 0.003 | 0 | 12 | n.m. | 1.03* | 1.69 | <0.0004 | 0.26 | 10.41 |
| 0.8 | 0.01 | 0 | 12 | n.m. | 1.09* | 1.88 | 0.0006 | 0.25 | 10.38 |
| 0.8 | 0.03 | 0 | 12 | n.m. | 1.07* | 1.59 | 0.0004 | 0.34 | 10.53 |
| 0.8 | 0.1 | 0 | 12 | n.m. | 1.14* | 1.56 | 0.0011 | 0.43 | 10.63 |
| 0.8 | 0.2 | 0 | 12 | n.m. | 1.29* | 2.16 | 0.0008 | 0.35 | 10.54 |
| 0.8 | 0 | 0.1 | 3 | 60 | 0.04 | 3.47 | <0.0004 | 33.1 | 12.52 |
| 0.8 | 0.001 | 0.1 | 3 | 70 | 0.05 | 1.91 | 0.026 | 37.2 | 12.57 |
| 0.8 | 0.003 | 0.1 | 3 | 60 | 0.04 | 2.69 | 0.024 | 35.5 | 12.55 |
| 0.8 | 0.01 | 0.1 | 3 | 60 | 0.04 | 2.29 | 0.055 | 36.3 | 12.56 |
| 0.8 | 0.03 | 0.1 | 3 | 65 | 0.06 | 1.74 | 0.15 | 36.3 | 12.56 |
| 0.8 | 0.05 | 0.1 | 3 | n.m. | <0.0002 | 8.38 | 0.15 | 33.2 | 12.52 |
| 0.8 | 0.1 | 0.1 | 3 | n.m. | <0.0002 | 3.46 | 0.56 | 29.9 | 12.48 |
| 0.8 | 0.15 | 0.1 | 3 | n.m. | <0.0002 | 5.85 | 0.68 | 22.0 | 12.34 |
| 0.8 | 0.2 | 0.1 | 3 | n.m. | <0.0002 | 9.15 | 0.34 | 24.0 | 12.38 |
| 0.8 | 0 | 0.1 | 12 | 58 | 0.14* | 2.19 | <0.0004 | 33.1 | 12.51 |
| 0.8 | 0.001 | 0.1 | 12 | 58 | 0.17* | 2.10 | 0.004 | 29.5 | 12.47 |
| 0.8 | 0.003 | 0.1 | 12 | 57 | 0.17* | 2.06 | 0.009 | 32.4 | 12.51 |
| 0.8 | 0.01 | 0.1 | 12 | 59 | 0.19* | 1.10 | 0.041 | 35.5 | 12.55 |
| 0.8 | 0.03 | 0.1 | 12 | 59 | 0.17* | 1.72 | 0.11 | 31.6 | 12.50 |
| 0.8 | 0.05 | 0.1 | 15 | n.m. | 0.04 | 1.83 | 0.22 | 48.0 | 12.68 |
| 0.8 | 0.1 | 0.1 | 15 | n.m. | 0.03 | 2.53 | 0.48 | 59.7 | 12.78 |
| 0.8 | 0.15 | 0.1 | 15 | n.m. | 0.01 | 4.60 | 0.54 | 51.7 | 12.71 |
| 0.8 | 0.2 | 0.1 | 15 | n.m. | 0.01 | 4.93 | 0.97 | 47.9 | 12.68 |
| 0.8 | 0 | 0.5 | 3 | 450 | 0.03 | 9.55 | <0.0004 | 166 | 13.22 |
| 0.8 | 0.001 | 0.5 | 3 | 460 | 0.03 | 11.7 | 0.021 | 166 | 13.22 |
| 0.8 | 0.003 | 0.5 | 3 | 460 | 0.04 | 5.61 | 0.048 | 166 | 13.22 |
| 0.8 | 0.01 | 0.5 | 3 | 460 | 0.04 | 6.45 | 0.17 | 158 | 13.20 |
| 0.8 | 0.03 | 0.5 | 3 | 480 | 0.05 | 26.1 | 0.57 | 162 | 13.21 |
| 0.8 | 0.05 | 0.5 | 3 | n.m. | <0.0002 | 2.14 | 0.17 | 169 | 13.23 |
| 0.8 | 0.1 | 0.5 | 3 | n.m. | <0.0002 | 3.83 | 1.96 | 147 | 13.17 |
| 0.8 | 0.15 | 0.5 | 3 | n.m. | <0.0002 | 14.3 | 2.89 | 122 | 13.09 |
| 0.8 | 0.2 | 0.5 | 3 | n.m. | <0.0002 | 17.6 | 3.44 | 132 | 13.12 |
| 0.8 | 0 | 0.5 | 12 | 400 | 1.26* | 3.46 | <0.0004 | 141 | 13.15 |
| 0.8 | 0.001 | 0.5 | 12 | 430 | 1.68* | 2.65 | 0.014 | 162 | 13.21 |
| 0.8 | 0.003 | 0.5 | 12 | 420 | 1.12* | 5.36 | 0.040 | 162 | 13.20 |
| 0.8 | 0.01 | 0.5 | 12 | 450 | 1.33* | 4.66 | 0.11 | 182 | 13.26 |
| 0.8 | 0.03 | 0.5 | 12 | 450 | 1.24* | 4.09 | 0.42 | 178 | 13.25 |
| 0.8 | 0.05 | 0.5 | 15 | n.m. | 0.03 | 2.56 | 0.69 | 409 | 13.61 |
| 0.8 | 0.1 | 0.5 | 15 | n.m. | 0.03 | 2.40 | 2.21 | 367 | 13.56 |
| 0.8 | 0.15 | 0.5 | 15 | n.m. | 0.01 | 10.9 | 3.51 | 313 | 13.50 |
| 0.8 | 0.2 | 0.5 | 15 | n.m. | 0.02 | 7.65 | 4.57 | 220 | 13.34 |
| 0.8 | 0 | 1 | 3 | 970 | 0.04 | 17.2 | <0.0004 | 309 | 13.49 |
| 0.8 | 0.001 | 1 | 3 | 1030 | 0.03 | 19.9 | 0.025 | 295 | 13.47 |
| 0.8 | 0.003 | 1 | 3 | 1050 | 0.04 | 23.8 | 0.097 | 309 | 13.49 |
| 0.8 | 0.01 | 1 | 3 | 980 | 0.03 | 18.7 | 0.30 | 316 | 13.50 |
| 0.8 | 0.03 | 1 | 3 | 1000 | 0.04 | 23.3 | 0.88 | 372 | 13.57 |
| 0.8 | 0.05 | 1 | 3 | n.m. | <0.0002 | 8.99 | 0.81 | 216 | 13.33 |
| 0.8 | 0.1 | 1 | 3 | n.m. | <0.0002 | 6.09 | 3.32 | 216 | 13.33 |
| 0.8 | 0.15 | 1 | 3 | n.m. | <0.0002 | 12.4 | 4.72 | 260 | 13.41 |
| 0.8 | 0.2 | 1 | 3 | n.m. | <0.0002 | 17.4 | 10.0 | 296 | 13.47 |
| 0.8 | 0 | 1 | 12 | 920 | 2.40* | 8.52 | <0.0004 | 263 | 13.42 |
| 0.8 | 0.001 | 1 | 12 | 960 | 2.57* | 7.85 | 0.021 | 302 | 13.47 |
| 0.8 | 0.003 | 1 | 12 | 910 | 2.44* | 8.07 | 0.072 | 316 | 13.50 |
| 0.8 | 0.01 | 1 | 12 | 900 | 2.52* | 8.73 | 0.22 | 309 | 13.49 |
| 0.8 | 0.03 | 1 | 12 | 1020 | 2.32* | 8.06 | 0.61 | 309 | 13.48 |
| 0.8 | 0.05 | 1 | 15 | n.m. | 0.02 | 8.80 | 2.92 | 469 | 13.67 |
| 0.8 | 0.1 | 1 | 15 | n.m. | 0.02 | 8.96 | 2.90 | 469 | 13.67 |
| 0.8 | 0.15 | 1 | 15 | n.m. | 0.03 | 9.19 | 5.71 | 652 | 13.81 |
| 0.8 | 0.2 | 1 | 15 | n.m. | 0.02 | 11.1 | 7.65 | 496 | 13.70 |

(n.m.: not measured; *: Ca concentrations for Ca/Si = 0.8 after 1 year equilibration are outliers.)

Appendix C. The calculated saturation indexes for different solid phases.

| Target Ca/Si | Target Al/Si | NaOH (M) | Time (months) | Strätlingite | Al(OH)_3_ | Katoite | CSHQ | Amorphous Silica | Gismondine | Chabazite | Sodalite |
| --- | --- | --- | --- | --- | --- | --- | --- | --- | --- | --- | --- |
| 0.8 | 0 | 0 | 3 | n.c. | n.c. | n.c. | -0.1 | -0.5 | n.c. | n.c. | n.c. |
| 0.8 | 0.001 | 0 | 3 | -3.4 | -0.6 | -15 | 0 | -0.5 | 3.7 | 5.3 | -88 |
| 0.8 | 0.003 | 0 | 3 | -3.5 | -0.5 | -15 | -0.1 | -0.5 | 3.7 | 5.4 | -87 |
| 0.8 | 0.01 | 0 | 3 | -2.5 | -0.5 | -13 | 0.2 | -0.5 | 4.3 | 5.9 | -86 |
| 0.8 | 0.03 | 0 | 3 | -2.8 | -0.5 | -14 | 0.2 | -0.5 | 4.2 | 5.9 | -86 |
| 0.8 | 0.1 | 0 | 3 | -2.9 | -0.6 | -14 | 0.1 | -0.6 | 3.9 | 5.4 | -86 |
| 0.8 | 0.2 | 0 | 3 | -2.3 | -0.5 | -13 | 0.3 | -0.5 | 4.4 | 6.0 | -85 |
| 0.8 | 0 | 0 | 12 | n.c. | n.c. | n.c. | 0.1 | -0.5 | n.c. | n.c. | n.c. |
| 0.8 | 0.001 | 0 | 12 | n.c. | n.c. | n.c. | 0 | -0.6 | n.c. | n.c. | n.c. |
| 0.8 | 0.003 | 0 | 12 | n.c. | n.c. | n.c. | 0 | -0.7 | n.c. | n.c. | n.c. |
| 0.8 | 0.01 | 0 | 12 | -5.4 | -1.8 | -16 | 0.1 | -0.6 | 1.4 | 2.8 | -95 |
| 0.8 | 0.03 | 0 | 12 | -5.7 | -2.1 | -16 | 0.1 | -0.8 | 0.6 | 1.7 | -97 |
| 0.8 | 0.1 | 0 | 12 | -4.7 | -1.8 | -15 | 0.1 | -0.9 | 1.3 | 2.2 | -95 |
| 0.8 | 0.2 | 0 | 12 | -4.8 | -1.8 | -15 | 0.2 | -0.7 | 1.5 | 2.8 | -94 |
| 0.8 | 0 | 0.1 | 3 | n.c. | n.c. | n.c. | -0.5 | -2.5 | n.c. | n.c. | n.c. |
| 0.8 | 0.001 | 0.1 | 3 | -4.6 | -2.4 | -11 | -0.4 | -2.8 | -2.1 | -5.2 | -8.8 |
| 0.8 | 0.003 | 0.1 | 3 | -4.9 | -2.4 | -12 | -0.4 | -2.7 | -2.1 | -4.7 | -8.5 |
| 0.8 | 0.01 | 0.1 | 3 | -4.1 | -2.1 | -11 | -0.5 | -2.7 | -1.4 | -4.2 | -6.7 |
| 0.8 | 0.03 | 0.1 | 3 | -2.9 | -1.6 | -9.3 | -0.4 | -2.8 | -0.5 | -3.6 | -4.6 |
| 0.8 | 0.05 | 0.1 | 3 | n.c. | -1.6 | n.c. | -1.0 | -2.1 | n.c. | n.c. | n.c. |
| 0.8 | 0.1 | 0.1 | 3 | n.c. | -1.0 | n.c. | -1.1 | -2.5 | n.c. | n.c. | n.c. |
| 0.8 | 0.15 | 0.1 | 3 | n.c. | -0.8 | n.c. | -1.0 | -2.1 | n.c. | n.c. | n.c. |
| 0.8 | 0.2 | 0.1 | 3 | n.c. | -1.1 | n.c. | -1.0 | -1.9 | n.c. | n.c. | n.c. |
| 0.8 | 0 | 0.1 | 12 | n.c. | n.c. | n.c. | -0.1 | -2.7 | n.c. | n.c. | n.c. |
| 0.8 | 0.001 | 0.1 | 12 | n.c. | n.c. | n.c. | -3.1 | -12.1 | n.c. | n.c. | n.c. |
| 0.8 | 0.003 | 0.1 | 12 | -7.0 | -3.0 | -13 | -0.1 | -2.7 | -2.3 | -5.1 | -12 |
| 0.8 | 0.01 | 0.1 | 12 | -2.9 | -2.2 | -8.5 | -0.1 | -3.1 | -1.4 | -5.0 | -9.6 |
| 0.8 | 0.03 | 0.1 | 12 | -2.2 | -1.7 | -8.4 | -0.1 | -2.8 | -0.2 | -3.2 | -5.6 |
| 0.8 | 0.05 | 0.1 | 15 | -3.0 | -1.6 | -9.3 | -0.5 | -3.0 | -0.8 | -4.1 | -3.0 |
| 0.8 | 0.1 | 0.1 | 15 | -2.7 | -1.4 | -9.1 | -0.5 | -3.0 | -0.4 | -3.7 | -0.7 |
| 0.8 | 0.15 | 0.1 | 15 | -3.7 | -1.2 | -11.3 | -0.7 | -2.6 | -0.2 | -2.8 | 1.6 |
| 0.8 | 0.2 | 0.1 | 15 | -3.2 | -1.0 | -10.9 | -0.7 | -2.6 | 0.4 | -2.1 | 3.5 |
| 0.8 | 0 | 0.5 | 3 | n.c. | n.c. | n.c. | -0.4 | -3.2 | n.c. | n.c. | n.c. |
| 0.8 | 0.001 | 0.5 | 3 | -6.1 | -3.3 | -12 | -0.3 | -3.1 | -4.2 | -7.8 | -4.3 |
| 0.8 | 0.003 | 0.5 | 3 | -5.0 | -2.9 | -10 | -0.3 | -3.4 | -3.7 | -7.9 | -3.8 |
| 0.8 | 0.01 | 0.5 | 3 | -3.8 | -2.3 | -9.4 | -0.3 | -3.3 | -2.4 | -6.4 | -1.1 |
| 0.8 | 0.03 | 0.5 | 3 | -3.3 | -1.9 | -9.8 | -0.3 | -2.8 | -0.9 | -3.8 | 5.9 |
| 0.8 | 0.05 | 0.5 | 3 | n.c. | -2.3 | n.c. | -0.6 | -3.9 | n.c. | n.c. | n.c. |
| 0.8 | 0.1 | 0.5 | 3 | n.c. | -1.2 | n.c. | -0.6 | -3.5 | n.c. | n.c. | n.c. |
| 0.8 | 0.15 | 0.5 | 3 | n.c. | -1.0 | n.c. | -0.5 | -2.8 | n.c. | n.c. | n.c. |
| 0.8 | 0.2 | 0.5 | 3 | n.c. | -1.0 | n.c. | -0.5 | -2.8 | n.c. | n.c. | n.c. |
| 0.8 | 0 | 0.5 | 12 | n.c. | n.c. | n.c. | 0.8 | -3.6 | n.c. | n.c. | n.c. |
| 0.8 | 0.001 | 0.5 | 12 | -2.5 | -3.4 | -5.4 | 0.9 | -3.9 | -3.7 | -8.9 | -10 |
| 0.8 | 0.003 | 0.5 | 12 | -2.1 | -2.9 | -5.9 | 0.8 | -3.5 | -2.4 | -6.7 | -5.0 |
| 0.8 | 0.01 | 0.5 | 12 | -1.0 | -2.5 | -4.4 | 0.9 | -3.7 | -1.8 | -6.4 | -3.1 |
| 0.8 | 0.03 | 0.5 | 12 | 0.1 | -1.9 | -3.3 | 0.9 | -3.7 | -0.7 | -5.5 | 0.1 |
| 0.8 | 0.05 | 0.5 | 15 | -2.8 | -2.1 | -6.3 | -0.3 | -4.4 | -3.3 | -9.6 | -2.1 |
| 0.8 | 0.1 | 0.5 | 15 | -1.7 | -1.5 | -5.4 | -0.4 | -4.4 | -2.2 | -8.4 | 1.0 |
| 0.8 | 0.15 | 0.5 | 15 | -2.7 | -1.3 | -8.2 | -0.4 | -3.6 | -1.3 | -6.0 | 6.4 |
| 0.8 | 0.2 | 0.5 | 15 | -1.7 | -1.0 | -7.2 | -0.3 | -3.5 | -0.4 | -4.8 | 7.8 |
| 0.8 | 0 | 1 | 3 | n.c. | n.c. | n.c. | -0.1 | -3.5 | n.c. | n.c. | n.c. |
| 0.8 | 0.001 | 1 | 3 | -6.3 | -3.6 | -12 | -0.1 | -3.4 | -5.0 | -9.1 | -2.9 |
| 0.8 | 0.003 | 1 | 3 | -5.1 | -3.0 | -10 | -0.1 | -3.3 | -3.8 | -7.8 | 0.8 |
| 0.8 | 0.01 | 1 | 3 | -4.2 | -2.5 | -9.6 | -0.1 | -3.5 | -3.0 | -7.3 | 3.0 |
| 0.8 | 0.03 | 1 | 3 | -3.2 | -2.1 | -8.4 | -0.1 | -3.5 | -2.2 | -6.6 | 5.5 |
| 0.8 | 0.05 | 1 | 3 | n.c. | -1.8 | n.c. | -0.4 | -3.5 | n.c. | n.c. | n.c. |
| 0.8 | 0.1 | 1 | 3 | n.c. | -1.1 | n.c. | -0.4 | -3.7 | n.c. | n.c. | n.c. |
| 0.8 | 0.15 | 1 | 3 | n.c. | -1.1 | n.c. | -0.3 | -3.5 | n.c. | n.c. | n.c. |
| 0.8 | 0.2 | 1 | 3 | n.c. | -0.9 | n.c. | -0.3 | -3.0 | n.c. | n.c. | n.c. |
| 0.8 | 0 | 1 | 12 | n.c. | n.c. | n.c. | 1.3 | -3.8 | n.c. | n.c. | n.c. |
| 0.8 | 0.001 | 1 | 12 | -2.1 | -3.5 | -4.7 | 1.3 | -3.9 | -3.6 | -8.8 | -6.0 |
| 0.8 | 0.003 | 1 | 12 | -1.1 | -3.0 | -3.7 | 1.3 | -3.9 | -2.7 | -7.9 | -3.1 |
| 0.8 | 0.01 | 1 | 12 | -0.1 | -2.5 | -2.8 | 1.3 | -3.9 | -1.6 | -6.7 | 0 |
| 0.8 | 0.03 | 1 | 12 | 0.7 | -2.1 | -1.9 | 1.3 | -3.9 | -0.8 | -6.0 | 3.0 |
| 0.8 | 0.05 | 1 | 15 | -2.2 | -1.6 | -6.5 | -0.2 | -4.1 | -2.1 | -7.7 | 5.8 |
| 0.8 | 0.1 | 1 | 15 | -2.2 | -1.6 | -6.5 | -0.2 | -4.1 | -2.1 | -7.7 | 5.8 |
| 0.8 | 0.15 | 1 | 15 | -1.3 | -1.4 | -4.9 | -0.2 | -4.4 | -1.9 | -8.0 | 6.1 |
| 0.8 | 0.2 | 1 | 15 | -1.4 | -1.2 | -5.7 | -0.2 | -4.1 | -1.3 | -6.9 | 8.3 |

(n.c.: not calculated as the Al and/or Ca concentrations were below the DL of ICP-MS and ICP-OES.)

Appendix D. The error calculations for solid phase composition.

| Target Ca/Si | Target Al/Si | NaOH (M) | Time (months) | Ca/Si in  C-A-S-H | Error of Ca/Si in C-A-S-H | Al/Si in  C-A-S-H | Error of Al/Si in C-A-S-H | *K_d_*  (m^3^/kg) | Error of  *K_d_* (m^3^/kg) |
| --- | --- | --- | --- | --- | --- | --- | --- | --- | --- |
| 0.8 | 0.001 | 0 | 3 | 0.80 | <0.0001 | 0.0010* | <0.00001 | 1.1 | <0.0001 |
| 0.8 | 0.003 | 0 | 3 | 0.81 | <0.0001 | 0.0030* | <0.00001 | 2.4 | <0.0001 |
| 0.8 | 0.01 | 0 | 3 | 0.80 | <0.0001 | 0.0100 | <0.00001 | 6.6 | <0.0001 |
| 0.8 | 0.03 | 0 | 3 | 0.79 | 0.002 | 0.0160 | 0.001 | 10.5 | 0.90 |
| 0.8 | 0.1 | 0 | 3 | 0.80 | 0.001 | 0.0911 | 0.001 | 54.3 | 0.62 |
| 0.8 | 0.2 | 0 | 3 | 0.79 | 0.002 | 0.1686 | 0.003 | 73.8 | 1.5 |
| 0.8 | 0.001 | 0 | 12 | 0.80 | <0.0001 | 0.0010* | <0.00001 | n.m. | <0.0001 |
| 0.8 | 0.003 | 0 | 12 | 0.80 | <0.0001 | 0.0030* | <0.00001 | n.m. | <0.0001 |
| 0.8 | 0.01 | 0 | 12 | 0.80 | <0.0001 | 0.0100 | <0.00001 | 170 | <0.0001 |
| 0.8 | 0.03 | 0 | 12 | 0.80 | <0.0001 | 0.0300 | <0.00001 | 760 | <0.0001 |
| 0.8 | 0.1 | 0 | 12 | 0.80 | <0.0001 | 0.1008 | <0.00001 | 845 | <0.0001 |
| 0.8 | 0.2 | 0 | 12 | 0.78 | 0.002 | 0.1864 | 0.002 | 2053 | 17.8 |
| 0.8 | 0.001 | 0.1 | 3 | 0.81 | <0.0001 | 0.0009 | <0.00001 | 0.33 | <0.0001 |
| 0.8 | 0.003 | 0.1 | 3 | 0.81 | <0.0001 | 0.0029 | <0.00001 | 1.1 | <0.0001 |
| 0.8 | 0.01 | 0.1 | 3 | 0.81 | <0.0001 | 0.0098 | <0.00001 | 1.7 | <0.0001 |
| 0.8 | 0.03 | 0.1 | 3 | 0.79 | 0.001 | 0.0295 | <0.00001 | 1.9 | <0.0001 |
| 0.8 | 0.05 | 0.1 | 3 | 0.82 | 0.001 | 0.051 | 0.0005 | 3.0 | <0.0001 |
| 0.8 | 0.1 | 0.1 | 3 | 0.79 | 0.003 | 0.094 | 0.0006 | 1.5 | 0.008 |
| 0.8 | 0.15 | 0.1 | 3 | 0.79 | 0.003 | 0.145 | 0.002 | 1.8 | 0.008 |
| 0.8 | 0.2 | 0.1 | 3 | 0.81 | 0.003 | 0.192 | 0.0002 | 4.7 | 0.041 |
| 0.8 | 0.001 | 0.1 | 12 | 0.81 | <0.0001 | 0.0010 | <0.00001 | 2.2 | <0.0001 |
| 0.8 | 0.003 | 0.1 | 12 | 0.81 | <0.0001 | 0.0030 | <0.00001 | 3.3 | <0.0001 |
| 0.8 | 0.01 | 0.1 | 12 | 0.80 | <0.0001 | 0.0099 | <0.00001 | 2.3 | <0.0001 |
| 0.8 | 0.03 | 0.1 | 12 | 0.81 | <0.0001 | 0.0297 | <0.00001 | 2.6 | <0.0001 |
| 0.8 | 0.05 | 0.1 | 15 | 0.80 | 0.0007 | 0.049 | <0.00001 | 2.0 | <0.0001 |
| 0.8 | 0.1 | 0.1 | 15 | 0.79 | 0.002 | 0.093 | 0.0006 | 1.7 | 0.012 |
| 0.8 | 0.15 | 0.1 | 15 | 0.78 | 0.004 | 0.139 | 0.001 | 2.2 | 0.021 |
| 0.8 | 0.2 | 0.1 | 15 | 0.78 | 0.005 | 0.174 | 0.003 | 1.5 | 0.024 |
| 0.8 | 0.001 | 0.5 | 3 | 0.84 | <0.0001 | 0.0010 | <0.00001 | 0.42 | <0.0001 |
| 0.8 | 0.003 | 0.5 | 3 | 0.82 | <0.0001 | 0.0028 | <0.00001 | 0.55 | <0.0001 |
| 0.8 | 0.01 | 0.5 | 3 | 0.82 | <0.0001 | 0.0095 | <0.00001 | 0.50 | <0.0001 |
| 0.8 | 0.03 | 0.5 | 3 | 0.86 | 0.004 | 0.0312 | <0.00001 | 0.45 | <0.0001 |
| 0.8 | 0.05 | 0.5 | 3 | 0.78 | 0.003 | 0.048 | 0.0002 | 2.6 | 0.01 |
| 0.8 | 0.1 | 0.5 | 3 | 0.79 | 0.002 | 0.092 | <0.00001 | 0.42 | <0.0001 |
| 0.8 | 0.15 | 0.5 | 3 | 0.83 | 0.003 | 0.138 | 0.0008 | 0.39 | 0.002 |
| 0.8 | 0.2 | 0.5 | 3 | 0.80 | 0.003 | 0.179 | 0.001 | 0.43 | 0.003 |
| 0.8 | 0.001 | 0.5 | 12 | 0.81 | <0.0001 | 0.0009 | <0.00001 | 0.62 | <0.0001 |
| 0.8 | 0.003 | 0.5 | 12 | 0.82 | <0.0001 | 0.0029 | <0.00001 | 0.67 | <0.0001 |
| 0.8 | 0.01 | 0.5 | 12 | 0.81 | <0.0001 | 0.0097 | <0.00001 | 0.81 | <0.0001 |
| 0.8 | 0.03 | 0.5 | 12 | 0.81 | <0.0001 | 0.0286 | <0.00001 | 0.63 | <0.0001 |
| 0.8 | 0.05 | 0.5 | 15 | 0.81 | <0.0001 | 0.047 | <0.00001 | 0.63 | <0.0001 |
| 0.8 | 0.1 | 0.5 | 15 | 0.80 | 0.0008 | 0.090 | <0.00001 | 0.37 | <0.0001 |
| 0.8 | 0.15 | 0.5 | 15 | 0.84 | 0.0007 | 0.136 | 0.0004 | 0.33 | 0.001 |
| 0.8 | 0.2 | 0.5 | 15 | 0.83 | 0.0005 | 0.181 | 0.0003 | 0.33 | 0.001 |
| 0.8 | 0.001 | 1 | 3 | 0.88 | <0.0001 | 0.0010 | <0.00001 | 0.33 | <0.0001 |
| 0.8 | 0.003 | 1 | 3 | 0.90 | <0.0001 | 0.0029 | <0.00001 | 0.25 | <0.0001 |
| 0.8 | 0.01 | 1 | 3 | 0.87 | <0.0001 | 0.0094 | <0.00001 | 0.27 | <0.0001 |
| 0.8 | 0.03 | 1 | 3 | 0.87 | 0.003 | 0.0290 | <0.00001 | 0.28 | <0.0001 |
| 0.8 | 0.05 | 1 | 3 | 0.81 | 0.004 | 0.048 | <0.00001 | 0.53 | <0.0001 |
| 0.8 | 0.1 | 1 | 3 | 0.79 | 0.003 | 0.086 | <0.00001 | 0.23 | <0.0001 |
| 0.8 | 0.15 | 1 | 3 | 0.82 | 0.003 | 0.135 | <0.00001 | 0.24 | <0.0001 |
| 0.8 | 0.2 | 1 | 3 | 0.84 | 0.003 | 0.161 | 0.0002 | 0.13 | <0.0001 |
| 0.8 | 0.001 | 1 | 12 | 0.82 | <0.0001 | 0.0009 | <0.00001 | 0.41 | <0.0001 |
| 0.8 | 0.003 | 1 | 12 | 0.82 | <0.0001 | 0.0028 | <0.00001 | 0.35 | <0.0001 |
| 0.8 | 0.01 | 1 | 12 | 0.83 | <0.0001 | 0.0094 | <0.00001 | 0.39 | <0.0001 |
| 0.8 | 0.03 | 1 | 12 | 0.82 | <0.0001 | 0.0281 | <0.00001 | 0.41 | <0.0001 |
| 0.8 | 0.05 | 1 | 15 | 0.84 | <0.0001 | 0.037 | <0.00001 | 0.11 | <0.0001 |
| 0.8 | 0.1 | 1 | 15 | 0.83 | 0.0009 | 0.089 | <0.00001 | 0.27 | <0.0001 |
| 0.8 | 0.15 | 1 | 15 | 0.83 | 0.001 | 0.128 | <0.00001 | 0.19 | <0.0001 |
| 0.8 | 0.2 | 1 | 15 | 0.84 | 0.0009 | 0.168 | 0.0002 | 0.18 | <0.0001 |

(*: Al concentrations in the solution are below the DL of ICP-OES and Al/Si ratios calculated considering Al concentration equals to zero.

Appendix E. The Al fraction in C-A-S-H for target Ca/Si = 0.8 in the absence of NaOH and presence of 0.1, 0.5 and 1 M NaOH after 3 months equilibration.


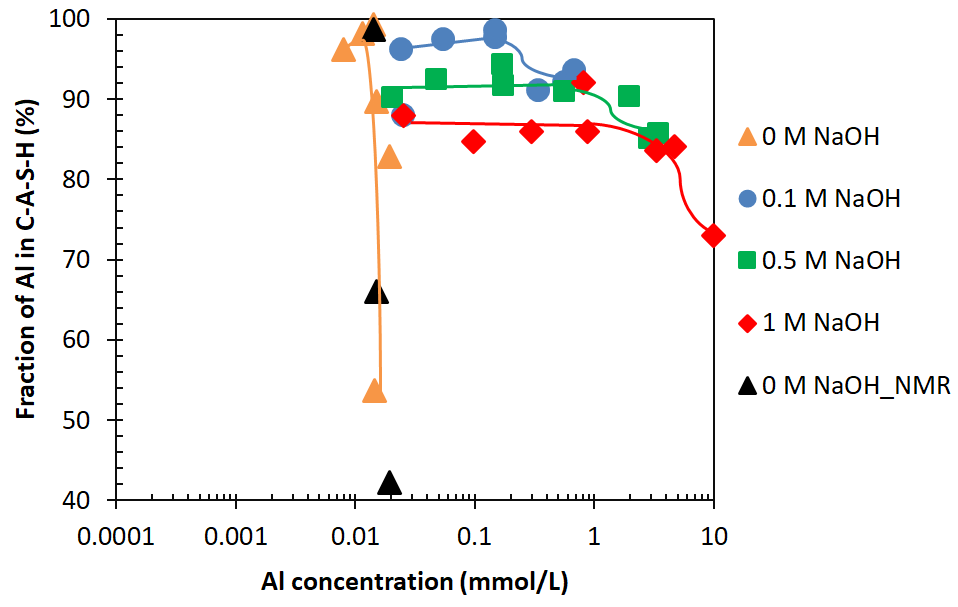


**Appendix F.** The Al/Si molar ratios and NaOH concentrations of C-A-S-H samples with target Ca/Si = 0.8 at 20 °C.

| **Target Ca/Si** | **Target Al/Si** | **NaOH (M)** | **Equilibration time (months)** |
| --- | --- | --- | --- |
|  | 0 |  |  |
|  | 0.001 |  |  |
|  | 0.003 |  |  |
|  | 0.01 |  |  |
| 0.8 | 0.03 | 0^a)^, 0.1, 0.5 and 1 | 3, 12^b)^ and 24^c)^ |
|  | 0.05 |  |  |
|  | 0.1 |  |  |
|  | 0.15 |  |  |
|  | 0.2 |  |  |

^a)^ samples without NaOH were not prepared at target Al/Si ratios of 0.05 and 0.15.

^b)^ samples at target Al/Si ratios of 0.05, 0.1, 0.15 and 0.2 in the presence of 0.1, 0.5 and 1 M NaOH were analyzed after 15 months instead of 12 months. ^c)^ Samples for NMR.

**Appendix G.** The fraction of Al in solution, C-A-S-H and different secondary phases at different NaOH concentrations and equilibration times for target Ca/Si = 0.8.

| NaOH (M) | Time (months) | Target Al/Si | % Al in  solution | % Al in  strätlingite | % Al in  Al(OH)_3_ | % Al in katoite | % Al in  C-A-S-H |
| --- | --- | --- | --- | --- | --- | --- | --- |
|  |  | 0.001 | 3.8 | 0 | 0 | 0 | 96.2 |
|  |  | 0.003 | 1.8 | 0 | 0 | 0 | 98.2 |
| 0 | 3 | 0.01 | 1.8 | 0 | 0 | 0 | 99.3 |
|  |  | 0.03 | 0.7 | 0 | 13.1 | 32.9 | 53.8 |
|  |  | 0.1 | 0.2 | 0 | 2.0 | 8.1 | 89.7 |
|  |  | 0.2 | 0.1 | 0 | 10.7 | 6.4 | 82.9 |
|  |  | 0.001 | ˂0.001 | 0 | 0 | 0 | 100 |
|  |  | 0.003 | ˂0.001 | 0 | 0 | 0 | 100 |
| 0 | 12 | 0.01 | 0.03 | 0 | 0 | 0 | 100 |
|  |  | 0.03 | 0.01 | 0 | 0 | 0 | 100 |
|  |  | 0.1 | 0.01 | 0 | 0 | 0 | 100 |
|  |  | 0.2 | ˂0.001 | 1.6 | 0 | 6.4 | 92.0 |
|  |  | 0.001 | 12.1 | 0 | 0 | 0 | 87.9 |
|  |  | 0.003 | 3.9 | 0 | 0 | 0 | 96.1 |
|  |  | 0.01 | 2.6 | 0 | 0 | 0 | 97.4 |
| 0.1 | 3 | 0.03 | 2.4 | 0 | 0 | 0 | 97.6 |
|  |  | 0.05 | 1.5 | 0 | 0 | 0 | 98.5 |
|  |  | 0.1 | 2.8 | 0 | 0 | 5.1 | 92.1 |
|  |  | 0.15 | 2.3 | 0 | 0 | 4.1 | 93.6 |
|  |  | 0.2 | 0.9 | 1.6 | 0 | 6.4 | 91.1 |
|  |  | 0.001 | 2.0 | 0 | 0 | 0 | 98.0 |
|  | 12 | 0.003 | 1.4 | 0 | 0 | 0 | 98.6 |
|  |  | 0.01 | 1.9 | 0 | 0 | 0 | 98.1 |
| 0.1 |  | 0.03 | 1.7 | 0 | 0 | 0 | 98.3 |
|  |  | 0.05 | 2.1 | 0 | 0 | 0 | 97.9 |
|  | 15 | 0.1 | 2.4 | 0 | 0 | 6.1 | 91.5 |
|  |  | 0.15 | 1.8 | 0 | 0 | 8.3 | 89.9 |
|  |  | 0.2 | 2.5 | 4.8 | 0 | 8.5 | 84.2 |
|  |  | 0.001 | 9.8 | 0 | 0 | 0 | 90.2 |
|  |  | 0.003 | 7.6 | 0 | 0 | 0 | 92.4 |
|  |  | 0.01 | 8.3 | 0 | 0 | 0 | 91.7 |
| 0.5 | 3 | 0.03 | 9.0 | 0 | 0 | 0 | 91.0 |
|  |  | 0.05 | 1.6 | 0 | 0 | 4.0 | 94.4 |
|  |  | 0.1 | 9.7 | 0 | 0 | 0 | 90.3 |
|  |  | 0.15 | 9.7 | 0 | 0 | 5.1 | 85.2 |
|  |  | 0.2 | 8.9 | 0 | 0 | 5.4 | 85.7 |
|  |  | 0.001 | 6.7 | 0 | 0 | 0 | 93.3 |
|  | 12 | 0.003 | 6.3 | 0 | 0 | 0 | 93.7 |
|  |  | 0.01 | 5.3 | 0 | 0 | 0 | 94.7 |
| 0.5 |  | 0.03 | 6.7 | 0 | 0 | 0 | 93.3 |
|  |  | 0.05 | 6.7 | 0 | 0 | 0 | 93.3 |
|  | 15 | 0.1 | 10.9 | 0 | 0 | 0 | 89.1 |
|  |  | 0.15 | 11.8 | 0 | 0 | 2.8 | 85.4 |
|  |  | 0.2 | 11.8 | 0 | 0 | 1.5 | 86.7 |
|  |  | 0.001 | 12.0 | 0 | 0 | 0 | 88.0 |
|  |  | 0.003 | 15.3 | 0 | 0 | 0 | 84.7 |
|  |  | 0.01 | 14.1 | 0 | 0 | 0 | 85.9 |
| 1 | 3 | 0.03 | 14.0 | 0 | 0 | 0 | 86.0 |
|  |  | 0.05 | 7.9 | 0 | 0 | 0 | 92.1 |
|  |  | 0.1 | 16.5 | 0 | 0 | 0 | 83.5 |
|  |  | 0.15 | 15.9 | 0 | 0 | 0 | 84.1 |
|  |  | 0.2 | 25.9 | 0 | 0 | 1.1 | 73.0 |
|  |  | 0.001 | 9.8 | 0 | 0 | 0 | 90.2 |
|  | 12 | 0.003 | 11.4 | 0 | 0 | 0 | 88.6 |
|  |  | 0.01 | 10.4 | 0 | 0 | 0 | 89.6 |
| 1 |  | 0.03 | 9.8 | 0 | 0 | 0 | 90.2 |
|  |  | 0.05 | 28.3 | 0 | 0 | 0 | 71.7 |
|  | 15 | 0.1 | 14.4 | 0 | 0 | 0 | 85.6 |
|  |  | 0.15 | 19.2 | 0 | 0 | 0 | 80.8 |
|  |  | 0.2 | 19.8 | 0 | 0 | 0.5 | 79.7 |

Appendix H. The effect of NaOH concentration on secondary phases' content for target Ca/Si = 0.8 with target Al/Si ratios of 0.03 and 0.2 after 3 months equilibration. The darkness of colors indicates an increase in NaOH concentration; 0 M NaOH is indicated by the dashed lines with light colors and 1 M NaOH represented by the solid lines with dark colors.


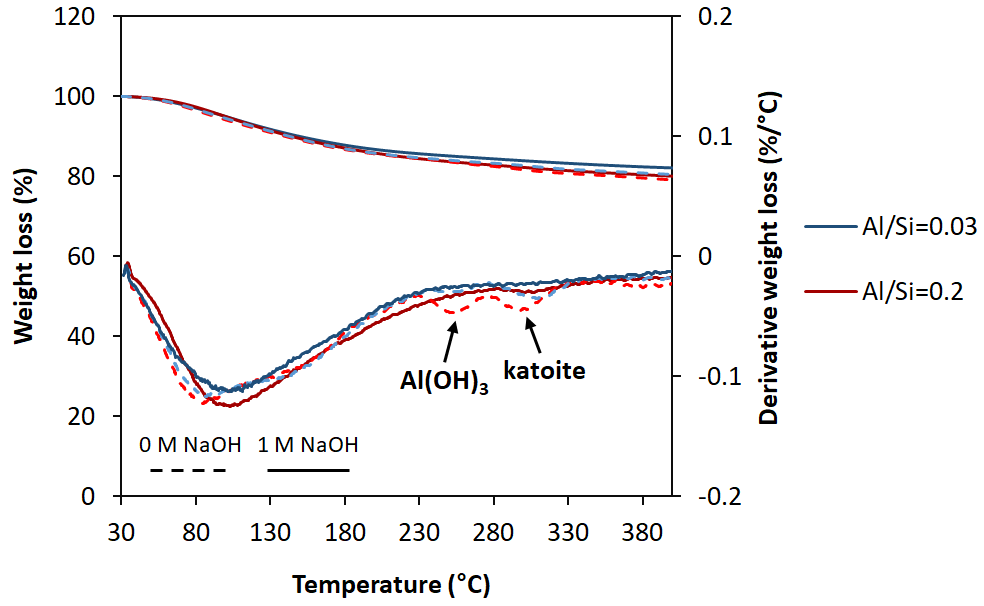

Supplement: Supplementary file 1 — Supplementary file1 (DOCX 283 KB) [file 11527_2022_2080_MOESM1_ESM.docx]
